# Mitochondrial fission is a critical modulator of mutant APP-induced neural toxicity

**DOI:** 10.1016/j.jbc.2021.100469

**Published:** 2021-02-25

**Authors:** Lauren Y. Shields, Huihui Li, Kevin Nguyen, Hwajin Kim, Zak Doric, Joseph H. Garcia, T. Michael Gill, Dominik Haddad, Keith Vossel, Meredith Calvert, Ken Nakamura

**Affiliations:** 1 Gladstone Institute of Neurological Disease, San Francisco, California, USA; 2 Graduate Programs in Neuroscience and Biomedical Sciences, University of California, San Francisco, San Francisco, California, USA; 3 Department of Neurology, University of California, San Francisco, San Francisco, California, USA

**Keywords:** Drp1, amyloid precursor protein (APP), mitochondria, mitochondrial calcium, mitochondrial fission, Alzheimer’s disease, neurodegeneration, neurodegenerative disease, 2DG, 2-deoxyglucose, Aβ, amyloid beta, AD, Alzheimer’s disease, APP, amyloid precursor protein, CCD, charge coupled device, cytCa^2+^, cytosolic calcium, Drp1, dynamin-related protein 1, Drp1cKO, hAPP mice that lack Drp1 in the CA1 and other forebrain neurons, EYFP-ER, EYFP targeted to the ER, hAPP, human amyloid precursor protein, MAMs, mitochondria-associated ER membranes, MCU, mitochondrial uniporter, mitoCa^2+^, mitochondrial Ca^2+^, MPTP, mitochondrial permeability transition pore, MWM, Morris water maze, NCLX, mitochondrial Na^+^/Ca^2+^ exchanger, PFA, 4% paraformaldehyde

## Abstract

Alterations in mitochondrial fission may contribute to the pathophysiology of several neurodegenerative diseases, including Alzheimer’s disease (AD). However, we understand very little about the normal functions of fission or how fission disruption may interact with AD-associated proteins to modulate pathogenesis. Here we show that loss of the central mitochondrial fission protein dynamin-related protein 1 (Drp1) in CA1 and other forebrain neurons markedly worsens the learning and memory of mice expressing mutant human amyloid precursor protein (hAPP) in neurons. In cultured neurons, Drp1KO and hAPP converge to produce mitochondrial Ca^2+^ (mitoCa^2+^) overload, despite decreasing mitochondria-associated ER membranes (MAMs) and cytosolic Ca^2+^. This mitoCa^2+^ overload occurs independently of ATP levels. These findings reveal a potential mechanism by which mitochondrial fission protects against hAPP-driven pathology.

Although the pathophysiology of Alzheimer’s disease (AD) remains poorly understood, several lines of evidence suggest that mitochondrial dysfunction and disrupted balance between mitochondrial fission and fusion may contribute to neurodegeneration (1). Indeed, levels of the central mitochondrial fission protein dynamin-related protein 1 (Drp1) are frequently increased in postmortem tissue from AD patients, while fusion proteins are decreased (2, 3), suggesting a shift toward fission. In addition, mutation or overexpression of a number of disease-related proteins (4, 5, 6, 7, 8), including amyloid precursor protein (APP) (9) and amyloid beta (Aβ) (10)*,* augment mitochondrial fragmentation, and Aβ has been proposed to cause toxicity by increasing function of Drp1 (10). Although it remains unclear whether increased fragmentation actually causes neurodegeneration, inhibiting Drp1 can protect against toxicity in models of Parkinson’s disease and Huntington’s disease (7, 8, 11, 12).

Importantly, disrupting the fission–fusion balance in either direction can be toxic, since insufficient mitochondrial fission produces excessive mitochondrial tubulation and also causes disease (1). Mutations in Drp1 are now recognized to cause a range of neurologic disorders from encephalopathy and neonatal lethality to refractory epilepsy (13, 14, 15, 16). In addition, Drp1 has been shown to lie downstream of the microtubule-associated protein tau, which functions to stabilize microtubules and actin, and also accumulates in intracellular aggregates in AD (17). Tau regulates Drp1 through effects on stabilizing actin (18), and tau can cause toxicity by blocking Drp1 localization to mitochondria (18). Therefore, the role of Drp1 in AD pathogenesis still remains unclear and may be context-dependent.

The functions of mitochondrial fission in healthy neurons also remain poorly understood, but appear to be multifactorial. Neurons require Drp1 to target mitochondria down distal axons (19, 20). In addition, neurons lacking Drp1 are unable to maintain normal levels of mitochondrial-derived ATP at the nerve terminal (21), at least in part due to intrinsic functional deficits in mitochondria lacking Drp1. Drp1 is also critical for respiration in other cells with high energy requirements such as cardiac myocytes (22), whereas the effect of Drp1 loss in other cell types, such as mouse embryonic fibroblasts (MEFs), is less consistent (21, 22, 23, 24, 25, 26, 27, 28). Fission is also widely hypothesized to support mitochondrial function by facilitating the turnover of dysfunctional mitochondria (29). Without fission, mitochondria may be too large to be engulfed by autophagosomes, causing dysfunctional mitochondria to accumulate (21, 23). However, mitochondrial fission may also support mitochondrial function more directly. For instance, mitochondrial fission by Drp1 occurs at the points of contact between mitochondria and the ER (mitochondria-associated membranes, MAMs (30)), raising the possibility that Drp1 influences MAM function and thus Ca^2+^ and lipid metabolism. Interestingly, cytosolic calcium (cytCa^2+^) promotes Drp1 recruitment to mitochondria through calcineurin and Ca^2+^/calmodulin-dependent protein kinase 1 alpha (31, 32), although it remains unclear how Drp1 ultimately impacts calcium homeostasis and whether any such effects are independent of its role in supporting ATP levels in neurons (21). Indeed, Ca^2+^ buffering may be particularly energetically expensive (33), and hence insufficient ATP might compromise the capacity of neurons to maintain normal cytosolic Ca^2+^ levels and gradients.

In order to better understand the functions of mitochondrial fission and how altered fission may influence the pathophysiology of AD, we examined the impact of loss of mitochondrial fission on the toxicity of mutant hAPP in mouse neurons.

## Results

### Loss of mitochondrial fission increases the toxicity of mutant APP *in vivo*

Increased mitochondrial fission has been proposed to underlie Aβ toxicity (34), and Drp1 inhibitors are protective in other mouse models of neurotoxicity (11, 35). To test if loss of the central mitochondrial fission protein Drp1 protects against the toxicity of mutant APP, we generated mice with targeted deletion of Drp1 on a mutant human APP background. First, Drp1^lox/lox^ mice were crossed with an Alzheimer’s mouse model, hAPP-J20 (36), referred to henceforth as hAPP, which expresses mutant hAPP (Swedish and Indiana mutations) in neurons under the PDGF-beta promoter. All hAPP mice were heterozygous carriers of the hAPP transgene. hAPP;Drp1^wt/lox^ mice were bred to homozygosity (hAPP;Drp1^lox/lox^). To generate hAPP mice that lack Drp1 in CA1 and other forebrain neurons (Drp1cKO), we bred CamKCre;Drp1^wt/lox^ and hAPP;Drp1^lox/lox^ mice to generate hAPP;Drp1^lox/lox^;CamKII-Cre mice. The resulting progeny (total n = 246 mice) was born in roughly Mendelian proportions including controls that lacked Cre (Drp1^wt/lox^ and Drp1^lox/lox^, 26.4%), Drp1 heterozygotes (Drp1^lox/wt^;CamKII-Cre,13.0%), Drp1cKO (Drp1^lox/lox^;CamKII-Cre, 16.7%), hAPP mice that lacked Cre (hAPP;Drp1^wt/lox^ and hAPP;Drp1^lox/lox^, 20.3%), hAPP Drp1 heterozygotes 13.8%, and hAPP Drp1cKO (9.75%).

**Figure 1:**
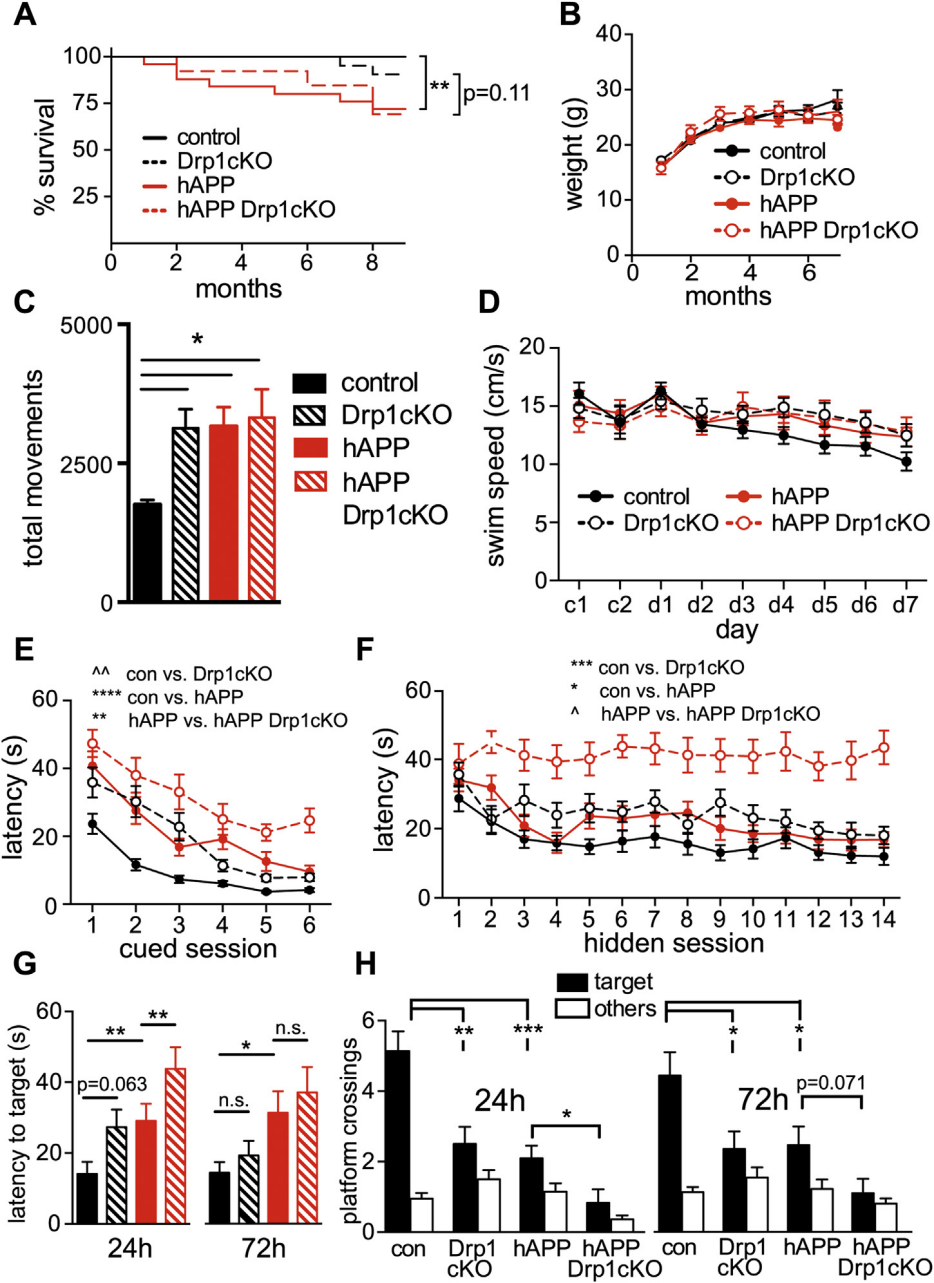
**Drp1 loss and hAPP expression combine to impair learning and memory**. *A*, hAPP (hAPP-J20;Drp1^wt/lox^ and hAPP-J20;Drp1^lox/lox^) mice had significant premature mortality (∗∗*p* < 0.01) compared with controls (Drp1^wt/lox^ and Drp1^lox/lox^); hAPP Drp1cKO (hAPP-J20;Drp1^lox/lox^;CamKII-Cre) mice had a trend (*p* = 0.11) toward premature mortality compared with Drp1cKO (Drp1^lox/lox^;CamKII-Cre), by log-rank Mantel–Cox test, n = 13 to 30 mice/group monitored from birth through 9 months of age. *B*, no weight differences were observed between genotypes up to 7 months. Data are means ± S.E.M.; n = 5-42 mice/group. *C*, 6–7-month-old Drp1cKO, hAPP, and hAPP Drp1cKO mice showed an increased number of total movements in an open field over the course of 15 min, as compared with controls (Drp1^wt/lox^ and Drp1^lox/lox^). Data are means ± S.E.M.; ∗*p* < 0.05 by one-way ANOVA and Holm–Sidak *post hoc* test, n = 9–12 mice/group. *D–F*, both procedural and spatial learning and memory were evaluated using the Morris water maze (MWM). *D*, no difference in swim speeds was found throughout the 2 days of procedural learning or 7 days of spatial learning by two-way ANOVA with repeated measures, indicating that all groups had intact motor function prior to the start of spatial training. *E*, procedural cued training conducted on the first 2 days over six sessions (c1-6) demonstrated significant but differential learning effects between the groups. Data are means ± S.E.M.; ∗∗*p* < 0.01, ∗∗∗∗*p* < 0.0001, ˆˆp<1e-10 by average rank latency with mixed-effect modeling, n = 12–22 mice/group. *F*, spatial learning and memory during hidden platform training in 6–7-month-old mice. Drp1cKO and hAPP showed significant learning impairments compared with Drp1WT (control). hAPP-J20 Drp1cKO (hAPP Drp1cKO) mice showed significant learning impairments compared with hAPP-J20 (hAPP) mice. Data are means ± S.E.M.; ∗*p* < 0.05, ∗∗∗*p* < 0.001, ˆp<1e-9 by average rank latency with mixed-effect modeling, n = 12 to 22 mice/group. *G* and *H*, spatial memory was evaluated using MWM probe trials at 24 and 72 h with the hidden platform removed and measured by latency to cross the former hidden platform location (target) (*G*) and number of platform location (target) and nontarget (other) crossings (*H*). Drp1cKO, hAPP, and hAPP Drp1cKO mice showed significant memory deficits. n = 12 to 22 mice/group. Data are means ± S.E.M.; n.s. = not significant, ∗*p* < 0.05, ∗∗*p* < 0.01, ∗∗∗*p* < 0.001 by Cox proportional hazards regression models (latency to cross) and Quasi-Poisson generalized linear models (platform crossings).

As expected, hAPP mice had decreased survival (Fig. 1*A*) (37). Loss of Drp1 expression did not affect the survival defect in hAPP mice, and all genotypes had similar body weights through 7 months of age (Fig. 1*B*). In open field tests, hAPP and hAPP Drp1cKO mice displayed increased movement in the open field (Fig. 1*C*), consistent with hyperactivity previously seen in hAPP mice (38) and indicating intact motor activity, as expected for the hAPP genotype. Notably, Drp1cKO mice also exhibited hyperactivity, a finding that could indicate a network hyperactivity similar to that found in hAPP mice (38). All genotypes exhibited similar swim speeds throughout procedural and spatial learning (Fig. 1*D*) and exhibited evidence of procedural learning during cued platform testing using the Morris water maze (MWM), indicating that there are no major sensorimotor deficits in any of the genotypes. Although procedural learning was attenuated in the hAPP and Drp1cKO groups, all groups except for hAPP Drp1cKO mice were equivalent in their procedural performance at the end of training (Fig. 1*E*). Rather than protecting against hAPP toxicity, Drp1KO markedly exacerbated the spatial learning and memory impairments of hAPP mice in the MWM. hAPP Drp1cKO mice were unable to learn the platform location despite training over 14 sessions during seven consecutive days (Fig. 1*F*), suggesting a functional synergism of Drp1 loss and hAPP *in vivo*. Drp1cKO and hAPP mice showed only subtle spatial learning deficits, based on rank order analysis of latency. No learning differences were noted with Drp1 heterozygotes (Fig. S1, *A* and *B*). Spatial learning and memory deficits of Drp1cKO and hAPP mice were confirmed during probe trial performance carried out 24 and 72 h after the last training trial. hAPP mice took significantly longer to cross over the former location of the hidden platform (target), and both Drp1cKO and hAPP mice did not cross its former location as frequently as control mice. hAPP Drp1cKO mice performed worse than mice harboring either mutation alone (Fig. 1, *G* and *H*). Therefore, complete Drp1 loss markedly worsens (rather than prevents) the adverse effects of hAPP on memory. In contrast, hAPP Drp1cHET mice had decreased latency to target at 72 h compared with hAPP mice and also crossed the former platform location more frequently than hAPP mice at 24 h. Therefore, partial Drp1 loss does not worsen spatial learning and memory and may actually protect against it (Fig. S1, *C* and *D*).

**Figure 2:**
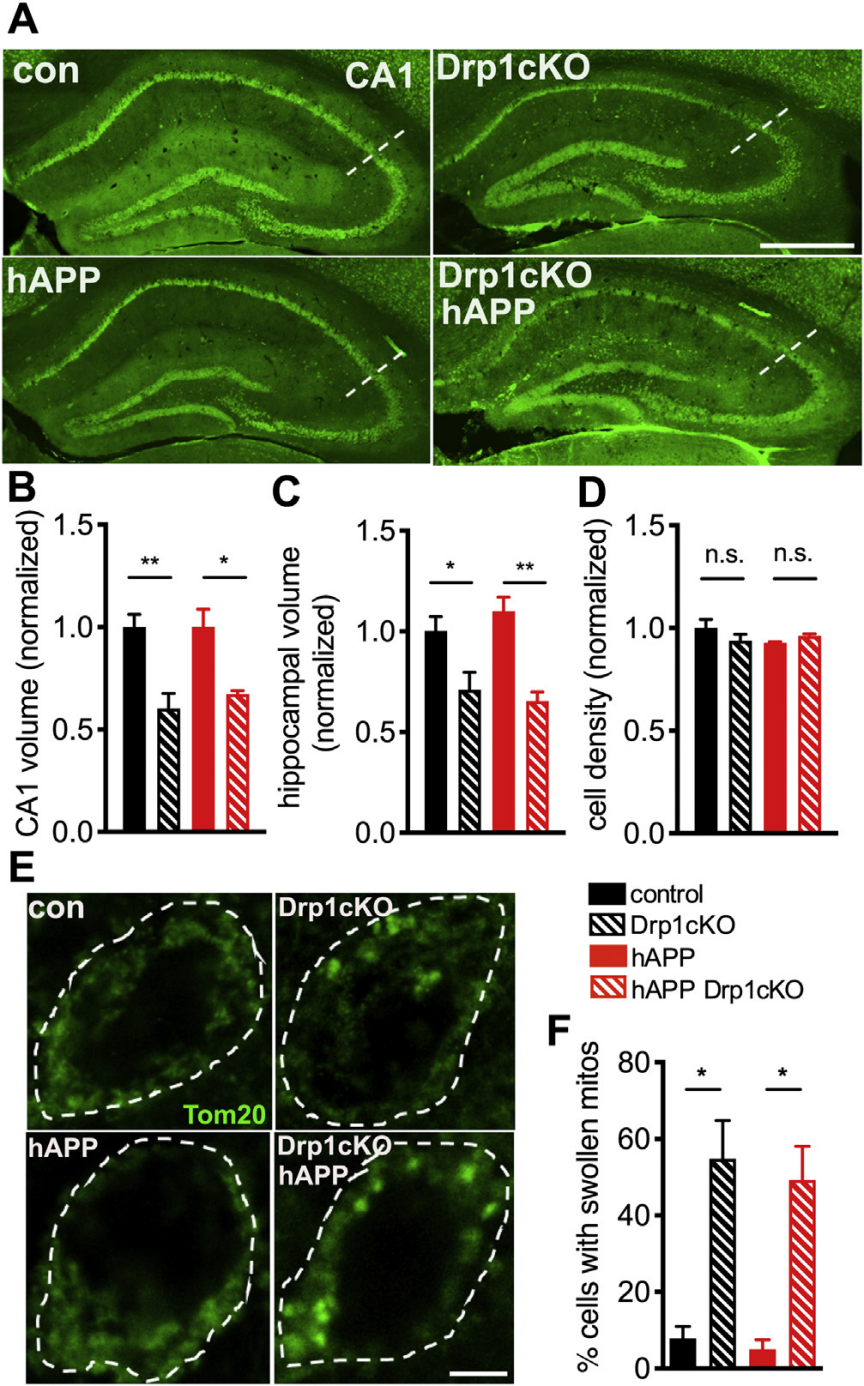
**hAPP does not exacerbate Drp1cKO-induced cell loss and morphologic changes.***A*, neuronal cell bodies labeled by NeuN staining in brain sections from 12-month-old mice. Hippocampi indicated by *dotted outlines*. Scale bar is 1 mm. *B* and *C*, Drp1 loss decreased both CA1 (*B*) and overall hippocampal (*C*) volume in 12-month-old mice. n = 4–5 mice/group (9–17 slices/mouse). Data are means ± S.E.M., ∗*p* < 0.05, ∗∗*p* < 0.01 by two-way ANOVA and Holm–Sidak *post hoc* test. *D*, Drp1cKO and hAPP Drp1cKO mice did not show any decrease in CA1 cell density at 12 months of age. n = 4–5 mice/group (4 slices/mouse). n.s. (not significant) by two-way ANOVA and Holm–Sidak *post hoc* test. *E*, Mitochondria in CA1 neurons in hippocampal slices from 6 to 7-month-old Drp1WT (control), Drp1cKO, hAPP-J20 (hAPP), and hAPP-J20 Drp1cKO (hAPP Drp1cKO) mice, identified by Tom20 immunofluorescence (*green*). Cell bodies (outer stippled outlines) were defined by Map2 staining. Scale bar is 4 μm. *F*, Drp1KO increased the proportion of cells with swollen mitochondria, while hAPP had no effect. n = 4 mice/group (3 slices/mouse). ∗*p* < 0.05 by Welch’s ANOVA and Games-Howell *post hoc* test (used instead of two-way ANOVA due to significant Levene’s test for equality of variance).

To determine whether Drp1 loss increased *in vivo* neurotoxicity by increasing Aβ levels, we examined Aβ deposition. However, concurrent loss of Drp1 did not significantly change the extent of age-dependent Aβ plaque deposition in hAPP mice, although the sensitivity of this experiment was limited by high variability (Fig. S2, *A* and *B*). Likewise, Drp1 loss alone did not change the level of murine APP in CA1 hippocampal neurons and also did not impact the total level of APP (murine and human) in hAPP Drp1cKO mice (Fig. S2, *C* and *D*). Moreover, hAPP expression alone did not affect hippocampal and CA1 volume and also did not contribute to the age-dependent loss of hippocampal or CA1 volume evident between 6-and 12-month-old Drp1cKO mice (Fig. 2, *A*–C, S3, *A* and *B*) (21) or affect CA1 cell density (Figs. 2*D* and S3*C*). Therefore, concurrent loss of Drp1 and hAPP did not increase the mild, age-dependent neuronal loss seen in Drp1cKO mice (21).

### Drp1 KO and hAPP converge to produce mitochondrial Ca^2+^ overload

We next investigated how loss of mitochondrial fission predisposes neurons to the toxicity of hAPP. Drp1KO neurons have swollen mitochondria, and tau may cause toxicity by producing excessive mitochondrial tubulation (18). However, hAPP alone had no impact on mitochondrial morphology or on Drp1 levels, nor did it affect the percent of cells with swollen mitochondria produced by Drp1cKO *in vivo* (Figs. 2, *E* and *F*, S4, *A* and *B*). In addition, neither hAPP nor Drp1cKO affected mitochondrial content in hippocampal neurons, as assessed by Tom20, PDH, and HSP60 immunofluorescence (Fig. 2*E*, and S4, *C–F*), indicating that a change in mitochondrial content is also unlikely to underlie the compound toxicity.

Mitochondria have critical functions in Ca^2+^ buffering, which influences both cytosolic and mitochondrial Ca^2+^ (mitoCa^2+^) levels (39, 40). Indeed, sufficient mitoCa^2+^ is required for respiratory chain enzyme function, but excessive Ca^2+^ can be toxic. To determine if Drp1KO and hAPP influence mitoCa^2+^ levels, we established a hippocampal neuron model system in which either Drp1 was deleted (Drp1KO), hAPP was overexpressed, or both. Specifically, we cotransfected Drp1^lox/lox^ primary hippocampal neurons with hAPP (41, 42) and either Cre recombinase (to remove Drp1) or a vector control, as well as Cepia3mt to measure mitoCa^2+^ (43) and mApple as a control to normalize for probe expression level. We confirmed hAPP expression (Fig. S5*A*) and that Cre expression led to the expected altered mitochondrial morphology and decreased Drp1 levels *in vitro*, indicative of Drp1 deletion (Figs. 2*E*, S4, *A*, *B* and *D*) (21).

**Figure 3:**
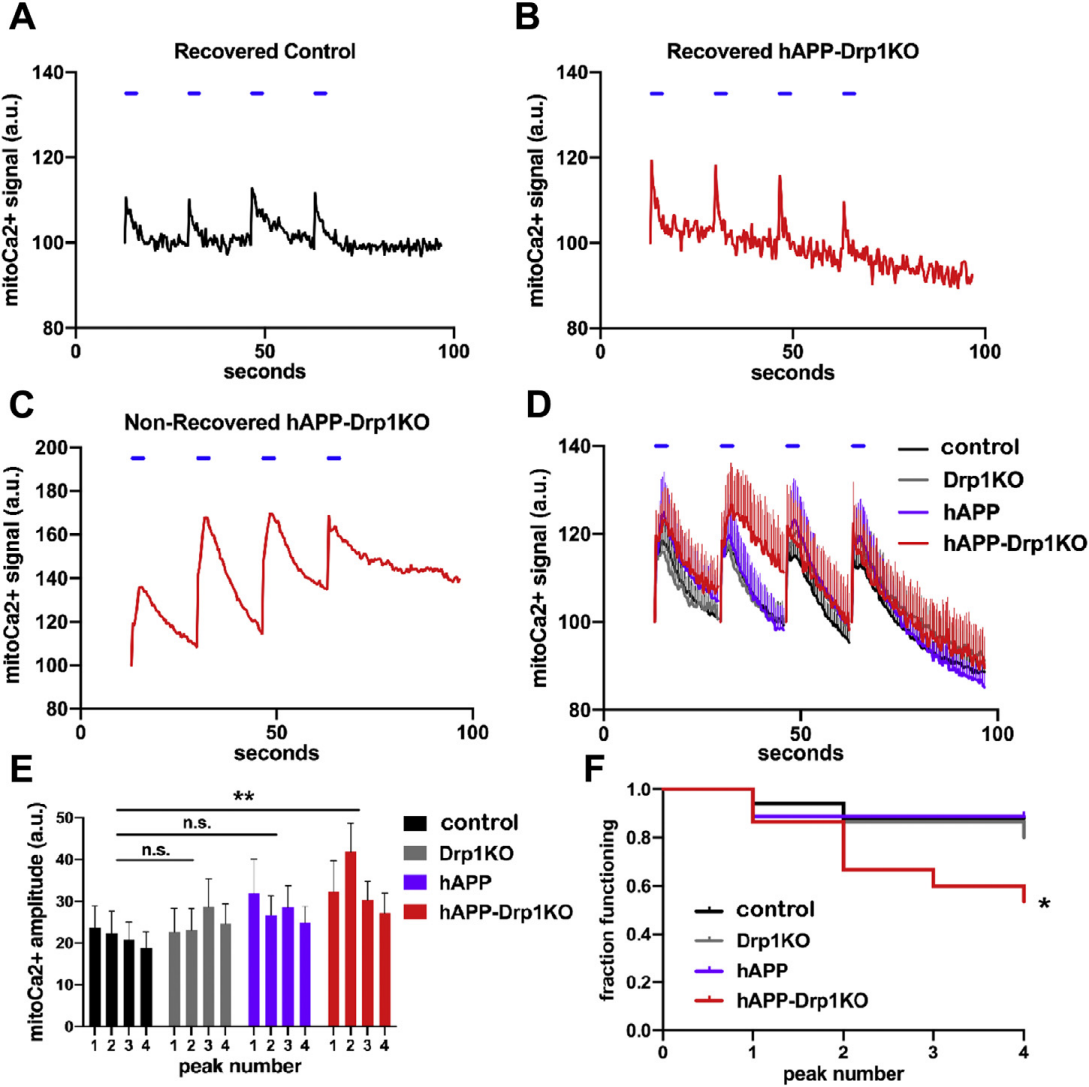
**hAPP and Drp1KO combine to overload mitochondria with calcium.** Primary hippocampal neurons from Drp1^lox/lox^ mice were cotransfected with Cre (to delete Drp1; Drp1KO), mutant hAPP, and/or control vector (control), as well as CEPIA3mt to visualize mitochondrial calcium (mitoCa^2+^) and mApple, and were subjected to a sequence of four individual electrical stimuli (30 Hz for 3s, *blue horizontal lines*) to evoke calcium entry. *A*, example trace of a control neuron that recovers baseline mitoCa^2+^ levels following each stimulus. *B* and *C*, Example traces of hAPP-Drp1KO neurons successfully (*B*) and unsuccessfully (*C*) recovering mitoCa^2+^ levels after evoked influx. *D*, average mitoCa^2+^ levels for control (*black*), Drp1KO (*gray*), hAPP (*purple*), and hAPP-Drp1KO (*red*) neurons. *E*, average amplitude for each mitoCa^2+^ peak in (*D*). The combination of hAPP and Drp1KO, but neither of the perturbations alone, increased mitoCa^2+^ loading during electrically evoked calcium entry compared to control. n = 15–18 coverslips/group (1 cell/coverslip), compilation of six independent experiments. Data show mean ± SEM; ∗∗*p* < 0.01 by two-way repeated measures ANOVA and Holm–Sidak *post hoc* test. *F*, graph shows the fraction of neurons from (*D*) that successfully recovered baseline mitoCa^2+^ levels following each of the four stimuli. The combination of hAPP and Drp1KO decreases the fraction of functional neurons; ∗*p* < 0.05 by log-rank test.

We first examined basal levels of mitoCa^2+^, estimated based on the ratio of basal Cepia3mt fluorescence/mApple fluorescence and found that they were similar in all groups (Fig. S5*B*). Next, we examined the neuron’s capacity to buffer Ca^2+^ during neural activity, using electrical field stimulation (30hz for 3s), which promotes preferential release of vesicles in the readily releasable pool (44) (Fig. 3, *A–D*, S5*C*). Following each electrical pulse train, mitoCa^2+^ transiently increased and then returned to baseline in most cells from all groups (*e.g.*, Fig. 3, *A* and *B*). However, in a subset of cells of all genotypes, mitoCa^2+^ levels failed to recover (*i.e.*, return to <30% peak amplitude) within ≈17s after electrical stimulation, defined as mitoCa^2+^ overload (*e.g.*, Fig. 3*C*). Overall, the combination hAPP-Drp1KO increased both the extent of mitoCa^2+^ influx (Fig. 3, *D* and *E*) and the frequency of mitoCa^2+^ overload (Fig. 3*F*), while neither hAPP nor Drp1KO alone affected these parameters. The increased mitoCa^2+^ influx was driven by increased mitoCa^2+^ influx into those cells that underwent mitoCa^2+^ overload, since mitoCa^2+^ influx was unchanged among those cells that recovered (Fig. S5, *C* and *D*). Moreover, mitoCa^2+^ overload may be triggered by excessive mitoCa^2+^ influx not decreased efflux. Consistent with this, among cells that recovered, both Drp1KO and hAPP-Drp1KO actually had a shorter half-life of mitoCa^2+^ decay (Fig. S5*E*), raising the possibility that loss of Drp1 upregulates mitoCa^2+^ efflux, for instance, to cope with increased import, as was observed in hAPP-Drp1KO cells. However, in hAPP-Drp1KO cells, our data suggest that increased mitoCa^2+^ influx eventually exceeds the capacity for mitoCa^2+^ export, resulting in mitoCa^2+^ overload, although the underlying mechanisms including the impact of Drp1KO and hAPP on mitoCa^2+^ influx require further investigation. This process is seemingly independent of Ca^2+^ efflux from the ER, as caffeine decreased ER Ca^2+^ levels similarly in all groups (Fig. S5*F*). Notably, neither Drp1KO nor hAPP significantly impacted levels of the mitochondrial uniporter (MCU) in CA1 neurons (Fig. S6, *A–D*).

### Drp1KO disrupts MAMs, but this does not underlie changes in mitoCa^2+^

We next investigated how hAPP and Drp1KO converge to disrupt mitoCa^2+^. We first focused on MAMs, which play a critical role in regulating calcium dynamics and transfer from the ER to the mitochondria (45). Interestingly, Aβ, mutant presenilins, and apoE4 can all increase the number, function, and/or content of MAMs (46, 47, 48). In addition, Presenilin-1 and -2 (catalytic subunits of γ-secretase) and APP are all enriched in the MAM fraction of cells (49). These studies suggest a role for MAM dysfunction in AD pathogenesis. In addition, Drp1 function may be implicated in MAMs; although MAMs can form without Drp1 present (30), Drp1 is recruited to MAMs prior to mitochondrial fission (30).

**Figure 4:**
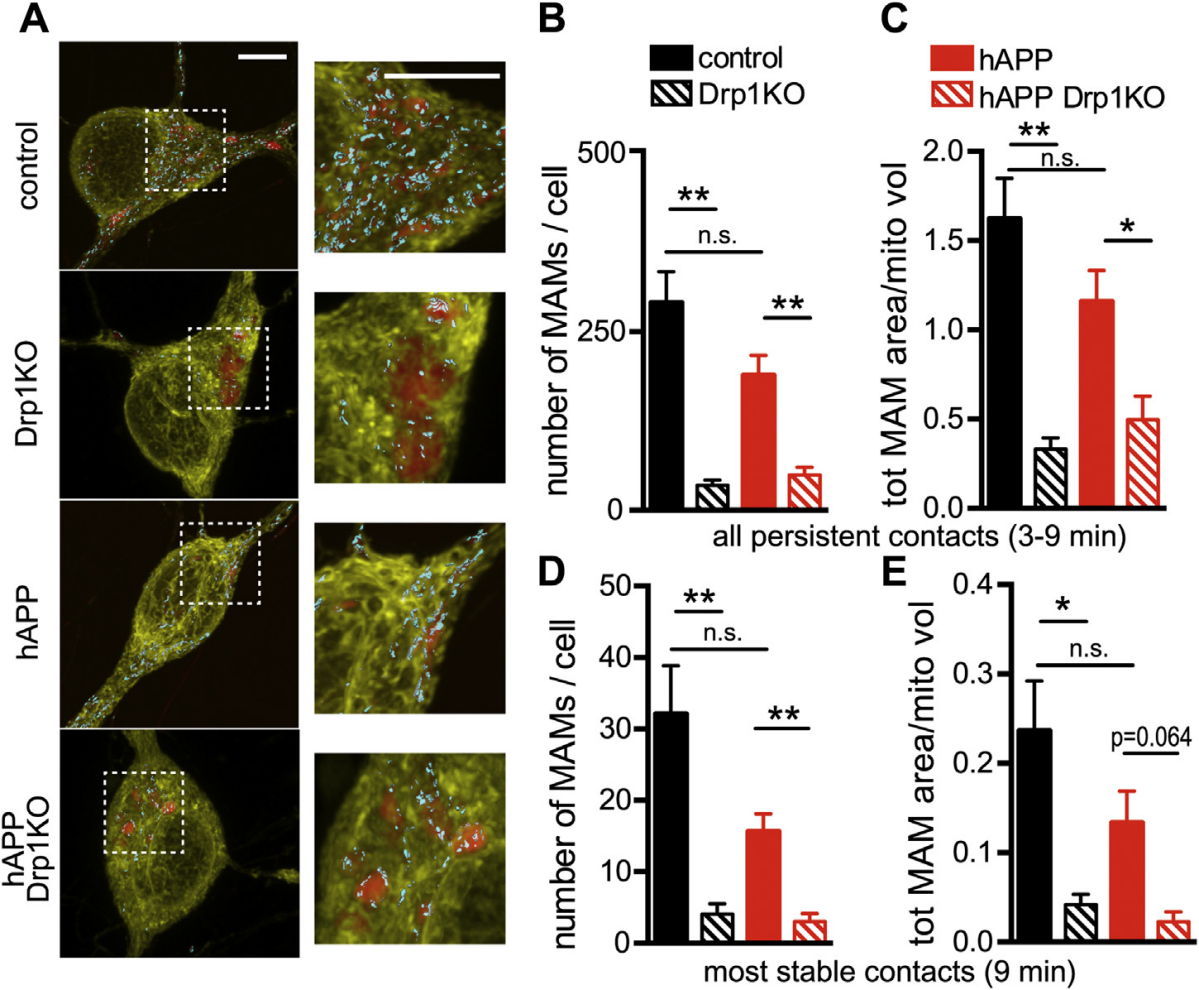
**Drp1 loss decreases MAMs in cultured neurons.** Drp1KO and control neurons, with or without mutant hAPP, were cotransfected with reporters to visualize the ER (*yellow*, eYFP-ER) and mitochondria (*red*, mitoFarRed). *A*, three-dimensional reconstructions of confocal Z-stacks (rendered *via* max projection) showing neuronal cell bodies with MAMs identified by areas showing ER-mitochondria colocalization (*cyan*; with surface rendering). *B*, in the presence or absence of hAPP, Drp1KO cells showed fewer persistent MAMs (defined as contacts lasting 3–5, 6–8, or 9 min) than Drp1WT cells. *C*, Drp1KO decreased the total area of ER–mitochondria contacts (normalized to total mitochondrial volume) with or without hAPP. *D* and *E*, in the most stable contacts (lasting 9 min), Drp1KO reduced MAM number and total MAM area in the presence and absence of hAPP expression. *(B–E),* hAPP alone had no significant effect on MAMs as compared with control. n = 8–11 coverslips/group (with 11–12 cells/group), compilation of three experiments. Data show mean ± SEM; *p* = 0.064, ∗*p* < 0.05, ∗∗*p* < 0.01, n.s. (not significant), by Welch’s ANOVA and Games-Howell *post hoc* test. Scale bars are 5 μm.

Considering that Aβ has also been proposed to mediate toxicity by increasing Drp1 function (10), and given Drp1’s critical role in regulating mitochondrial morphology (20, 23, 44), we hypothesized that Drp1KO may prevent the ability of mutant APP to alter the number, structure, or function of MAMs. We created three-dimensional reconstructions of confocal Z-stacks showing neuronal cell bodies from control and Drp1KO cells expressing EYFP targeted to the ER (EYFP-ER, yellow) and mito-FarRed (red) and identified contact regions as areas with persistent colocalization of the two probes for >3 min (50). Drp1KO neurons showed significantly fewer persistent MAMs and smaller MAM area per mitochondrial content (Fig. 4, *A-E*). This finding that Drp1KO neurons have fewer MAMs was supported by decreased colocalization of the MAM marker sigma-1 receptor (47) with mitochondria and confirmed by electron microscopy (Figs. S7, *A–C*, S8, *A and B*). However, hAPP did not alter the Drp1KO-induced decrease in the number and size of MAMs nor sigma-1 receptor colocalization with mitochondria (Figs. 4, *A–E*, and S7, *A–C*). Therefore, the effects of Drp1KO on MAM formation are not sufficient for the synthetic effect of Drp1KO and hAPP on mitoCa^2+^.

### Mitochondrial Ca^2+^ overload is not caused by excessive cytCa^2+^

**Figure 5:**
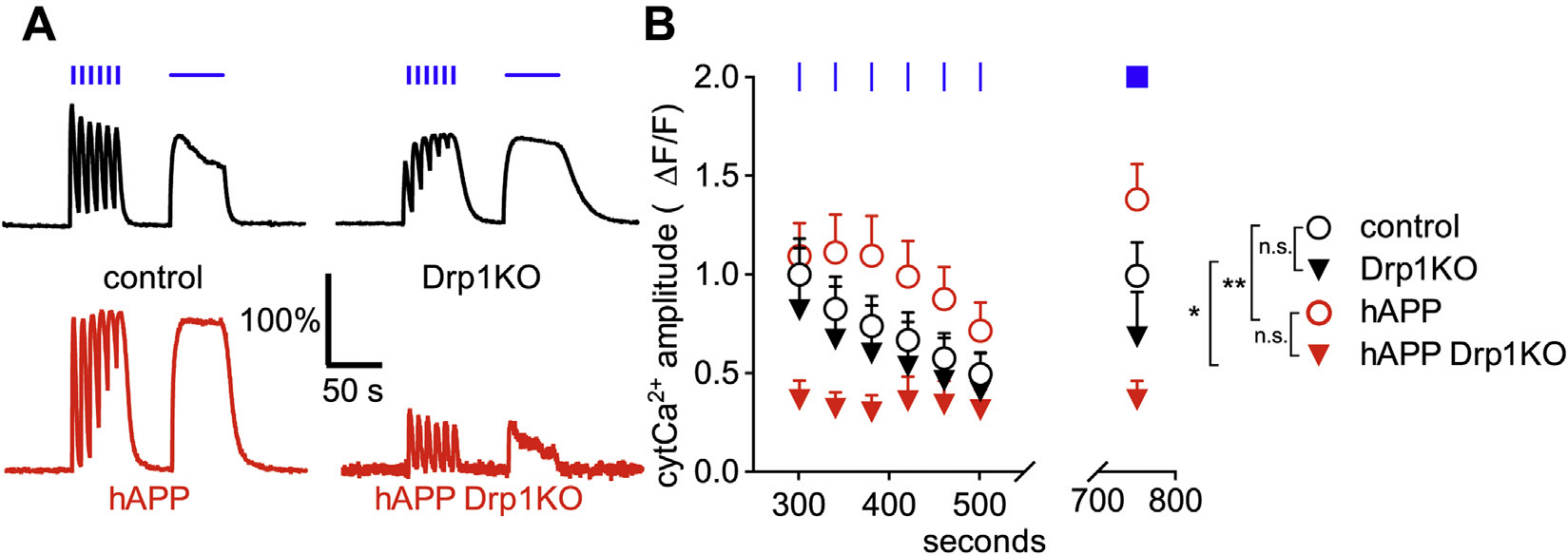
**hAPP expression increases evoked cytosolic calcium in the cell body of neurons, but drastically decreases evoked calcium in the absence of Drp1.***A* and *B*, Drp1KO and control neurons, with or without mutant hAPP, were cotransfected with the cytosolic calcium (cytCa^2+^) sensor GCaMP6f (81) and subjected to electrical stimulation (30 Hz for 3 s (*blue vertical bars*) and 10 Hz for 60 s (*horizontal blue bar*)). Drp1KO alone had no significant effect on the amplitude of evoked cytCa^2+^, whereas hAPP expression increased cytCa^2+^ in the presence of Drp1 and decreased cytCa^2+^ in the absence of Drp1. n = 7–8 coverslips/group (with 17–60 cells/group), compilation of three experiments. Data are representative traces normalized to baseline and control (*A*) and means ± S.E.M. (*B*) ∗*p* < 0.05 Drp1KO versus hAPP Drp1KO, ∗∗*p* < 0.01 control versus hAPP, ∗∗∗∗*p* < 0.0001 hAPP versus hAPP Drp1KO by two-way ANOVA and Holm–Sidak test. Control versus Drp1KO was not significant (n.s.).

Considering that Drp1KO disrupts MAMs, we asked whether the mitoCa^2+^ overload in hAPP-Drp1KO neurons is driven by excessive mitoCa^2+^ import from the cytosol, rather than the ER. To distinguish between an intrinsic increase in Ca^2+^ transport into mitochondria and increased mitoCa^2+^ influx secondary to elevated cytCa^2+^, we examined cytCa^2+^ levels with GCaMP6f, a fluorescence-based calcium sensor with high temporal resolution (51). hAPP alone increased cytCa^2+^ versus control in response to electrical field stimulation (30hz for 3s and 10hz for 60s), consistent with prior work showing that mutant APP increases cytCa^2+^ levels following Ca^2+^ influx through the plasma membrane or from the ER (52). Drp1KO alone did not affect the extent of increase in cytCa^2+^ in response to electrical stimulation; however, the combination of hAPP and Drp1KO markedly decreased cytCa^2+^ (Fig. 5, *A* and *B*). Although the mechanism and significance of this compounding decrease in cytCa^2+^ are unclear, it shows that excessive cytCa^2+^ per se is not the cause of mitoCa^2+^ overload. Instead, our data suggest that the most likely mechanism driving mitoCa^2+^ overload in the hAPP-Drp1KO neurons is increased shuttling of cytCa^2+^ into the mitochondria, which in turn results in a deficit of cytCa^2+^.

### mitoCa^2+^ overload occurs independent of an effect of hAPP on ATP levels

**Figure 6:**
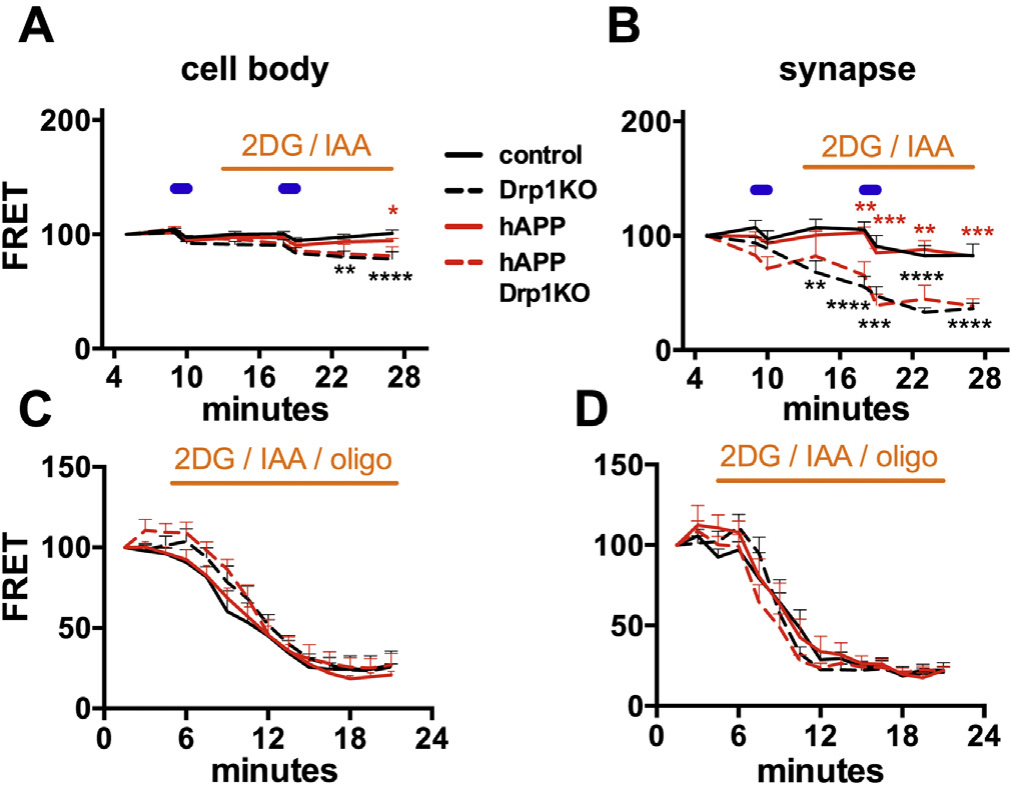
**Drp1KO, but not hAPP expression, reduces mitochondria-derived ATP at synapses more than at cell bodies**. Drp1KO and control neurons, with or without mutant hAPP, were cotransfected with an ATP-based FRET sensor (ATP1.03^YEMK^) (49). *A*, when forced to rely on mitochondria for ATP (acute absence of glucose, addition of glycolytic inhibitors 2-deoxyglucose (2DG) and iodoacetate (IAA); *orange horizontal bar*), Drp1KO neurons with or without hAPP had only slightly decreased ATP levels at the cell body after stimulation (10 Hz ∗ 60 s, *blue horizontal bars*). *B*, in contrast, Drp1KO neurons with or without hAPP had markedly reduced ATP levels at the synapse under these conditions. hAPP did not affect ATP levels. n = 6–12 coverslips/group (with 67–105 boutons and 15–22 cells per group), compilation of four experiments. *C* and *D*, To estimate basal ATP consumption, we simultaneously blocked glycolytic production with 2DG and IAA and respiration with oligomycin (oligo). Rates of consumption, assessed in the absence of electrical stimulation, did not differ across groups at the cell body (*C*) or the synapse (*D*), as indicated by the initial slope of decline in ATP level. n = 6–8 coverslips/group (with 56–68 boutons and 6–10 cells per group), compilation of three experiments. Data are means ± S.E.M.; ∗*p* < 0.05, ∗∗*p* < 0.01, ∗∗∗*p* < 0.001, ∗∗∗∗*p* < 0.0001 control versus Drp1KO (*black*) and hAPP versus hAPP Drp1KO (*red*) by two-way ANOVA with repeated measures and Holm–Sidak test.

We previously showed that neurons lacking Drp1 have decreased mitochondrial-derived ATP levels (21). hAPP can also impair respiratory function (53, 54), and optimal cytCa^2+^ and mitoCa^2+^ are required for efficient oxidative phosphorylation (39). To determine if hAPP converges with Drp1KO to produce energy failure, we cotransfected Drp1^lox/lox^ hippocampal neurons with a FRET-based ATP sensor (ATP1.03^YEMK^ (55)) and either Cre (to delete Drp1), or hAPP, or vector control. To specifically measure mitochondrial-derived ATP, we examined FRET levels in the acute absence of glucose and in the presence of glycolytic inhibitors 2-deoxyglucose (2DG) and iodoacetate (21, 44). As expected, under these conditions Drp1KO axons could not maintain ATP levels (21), and ATP levels at the cell body were also slightly but significantly decreased (Fig. 6, *A* and *B*, S9, *A* and *B*). However, hAPP expression alone did not cause mitochondrial ATP deficits at the cell body or synapse, and hAPP did not alter ATP levels in Drp1KO neurons, even upon electrical stimulation to increase energy demands.

Given the energetic requirement needed to maintain calcium gradients, disruptions in cytosolic and mitochondrial calcium dynamics could also reflect altered ATP consumption or could themselves lead to altered ATP consumption. To test if ATP consumption is impacted, we monitored the rate of decline in ATP levels after blocking all energy production using oligomycin (to inhibit respiration), 2DG and iodoacetate (to block glycolysis). The initial rate of decline in ATP soon after all ATP production is blocked, without electric stimulation, provided a surrogate for the basal rate of ATP consumption. However, neither Drp1KO nor hAPP affected the rate of ATP consumption (Fig. 6, *C* and *D*). Therefore, hAPP does not synergize with loss of Drp1 to disrupt mitochondrial-derived ATP *in vitro*. Instead, our data support that hAPP combines with a mitochondrial insult (here a change in mitochondrial fission) to disrupt mitochondrial Ca^2+^ homeostasis independent of energy levels, although this does not exclude the possibility that energy levels are ultimately affected *in vivo*.

## Discussion

Changes in mitochondrial fission have been implicated in the pathophysiology of AD, but we understand little about how the level of fission influences the toxicity of key AD proteins. Here, we show that loss of fission alone does not alter either cytosolic or mitochondrial Ca^2+^ homeostasis, but it converges with mutant hAPP to produce mitochondrial Ca^2+^ overload, seemingly independent of Drp1KO’s effect of decreasing ATP levels in neurons.

### Drp1 protects mitochondria against hAPP-induced mitochondrial Ca^2+^ overload

AD and other neurodegenerative diseases are multifactorial, but little is known regarding how contributing processes converge to produce toxicity. Here, we dissect how two stressors implicated in the pathophysiology of AD combine to produce toxicity. First, we show that the strong behavioral deficits produced by combining Drp1KO and hAPP *in vivo* cannot be attributed to changes in the mitochondrial morphology or content. This is surprising, since Drp1 has a primary role in regulating mitochondrial morphology (20, 21) and has been hypothesized to impact mitochondrial turnover (56). Instead, we show that loss of mitochondrial fission converges with hAPP to cause mitoCa^2+^ overload. Future studies are required to determine if hAPP predisposes to mitoCa^2+^ overload by increasing Aβ secretion from cells, or if other APP fragments may also contribute.

We hypothesized that the changes in mitochondrial Ca^2+^ might be explained by the effects of Drp1KO and hAPP on ER–mitochondria contacts. Indeed, Aβ, APOE4, and mutant presenilins can increase MAM formation (46, 47, 48) and Drp1 is recruited to MAMs (30). However, it has remained unclear if mitochondrial fission at these sites also plays a role in forming or maintaining these contacts. Here, we show that neurons require Drp1 to maintain MAMs, consistent with prior findings that inhibiting Drp1 decreases MAMs, while increasing mitochondrial fragmentation increases MAMs (57). The mechanism by which Drp1KO disrupts MAMs remains to be defined, but we hypothesize that steric factors contribute, especially given the characteristic swollen, rounded mitochondria at the cell body of Drp1KO neurons (20, 21). hAPP alone did not significantly affect the number of MAMs in our paradigm. This is in contrast with previous literature showing that Aβ increases MAMs (46, 47, 48). The basis for this discrepancy requires further investigation, but may relate to differences in the relative level and conformation of Aβ applied to the cells, as well as other potential functions of hAPP in the MAM fraction of cells (49). Although we cannot exclude the possibility that the Drp1KO-mediated decrease in MAM formation contributes to mitoCa^2+^ overload, hAPP did not amplify the effect on MAMs, suggesting that the compound effect of hAPP and Drp1KO on mitoCa^2+^ is not driven by a change in the number MAMs.

Alternatively, our data support a model in which Drp1KO promotes mitoCa^2+^ overload in hAPP-Drp1KO neurons by driving excessive influx of cytosolic Ca^2+^ into mitochondria. We speculate that increased mitoCa^2+^ is driven by the changes in mitochondrial shape, which may disrupt the relative capacity for mitoCa^2+^ import versus export, as proposed in a recent study showing that Drp1KO myofibers also have increased mitoCa^2+^ influx following electrical stimulation (58). However, increased mitoCa^2+^ may also cause changes in mitochondrial shape and degeneration. Indeed, toxic insults that increase cytCa^2+^ typically produce more fragmented mitochondria indicative of increased mitochondrial fission (59), and increasing mitoCa^2+^ by overexpressing the mitochondrial Ca^2+^ uniporter (MCU) drives mitochondrial fragmentation in neurons (60), while inhibiting MCU normalizes mitoCa^2+^ and protects against degeneration (58). Although it remains unclear how exactly mitochondrial fragmentation influences toxicity, our findings raise the possibility that mitochondrial fission is a protective mechanism that enables mitochondria to either prevent or cope with the excessive mitoCa^2+^ influx.

### hAPP-Drp1KO mitochondrial Ca^2+^ overload occurs independent of ATP

Our dissection of the hAPP-Drp1 interaction identified increases in mitoCa^2+^ that are seemingly independent of effects on mitochondrial-derived ATP. Although it is well recognized that mitochondria have functions such as Ca^2+^ homeostasis that are distinct from their roles in ATP production, these factors are often difficult to dissociate. Indeed, sufficient mitoCa^2+^ is required to maintain respiratory enzyme function (61, 62), whereas excessive mitoCa^2+^ depolarizes mitochondria, transiently (63) or continuously, by opening the permeability transition pore (MPTP) (64). However, here we show that mitoCa^2+^ was disrupted in hAPP-Drp1KO neurons in the absence of an additional disruption of mitochondrial-derived ATP levels beyond that observed in Drp1KO neurons. Although ATP levels may ultimately change *in vivo*, this suggests that changes in mitoCa^2+^ occur proximally, and are the primary or initiating event, rather than lying downstream of a change in ATP production or consumption. Nonetheless, further investigation is required to fully dissociate these parameters, as the effect of Drp1KO on decreasing ATP could still be necessary, but simply not sufficient for the combined effects of hAPP and Drp1 on mitoCa^2+^ dynamics. This might occur, for instance, if decreased mitochondrial ATP levels compromise mitochondrial Ca^2+^ efflux and thereby predispose cells to mitochondrial Ca^2+^ overload when faced with a second stressor such as hAPP. Sporadic AD is a multifactorial disorder, and although complicated, this type of mechanistic insight is necessary to begin to understand how distinct toxic insults may combine to drive neuronal dysfunction and degeneration and to provide insight into how such complicated interactions might be targeted therapeutically.

### Therapeutic potential of decreasing mitoCa^2+^ and Drp1 in AD

This work expands the field’s knowledge on the impact of Ca^2+^ homeostatic changes and mitochondrial function in models of AD. It is tempting to speculate that the same mitoCa^2+^ changes we observed in isolated neurons occur *in vivo* and might contribute to the compounding of learning and memory deficits in Drp1KO-hAPP mice. Indeed, mutations in APP and other proteins implicated in AD have been shown to disrupt cytosolic and ER calcium homeostasis (65, 66, 67), and Aß oligomers can increase mitoCa^2+^ levels, perhaps due to increased uptake secondary to increased expression of the mitoCa^2+^ uniporter (MCU) (68, 69). Interestingly, patients with AD have decreased expression of the mitochondrial Na^+^/Ca^2+^ exchanger (NCLX). Moreover, 3xTg-AD mice have impaired mitoCa^2+^ efflux, and rescuing mitoCa^2+^ efflux by expressing NCLX markedly improved behavioral deficits and Aß and tau pathology (70). However, further work is required to fully understand the impact of mitochondrial function and calcium dysfunction in AD and the possible therapeutic opportunities.

Lastly, our data suggest that Drp1 inhibition may be a risky target for AD therapeutics. While excessive Drp1 function has been hypothesized to mediate Aβ toxicity (34), Drp1KO not only fails to prevent hAPP toxicity, it actually markedly exacerbates it. While our findings are consistent with the possibility that partial Drp1 inhibition could be protective (2, 12), careful calibration of the fission–fusion balance may be required for therapeutic efficacy. Our data clearly support the hypothesis that fission–fusion balance is necessary to support cellular function and that an excessive shift in either direction can cause neuronal dysfunction (13, 14, 15, 16, 71, 72, 73).

## Experimental procedures

### Animals

Floxed Drp1 (23) and hAPP-J20 (36) mice have been described. CamKCre mice (74) were obtained from Jackson Laboratory. Mice were group-housed in a colony maintained with a standard 12 h light/dark cycle and given food and water *ad libitum*. Experiments were performed on age-matched mice of either sex. No differences between sexes were noted in any of the experiments. Experiments were conducted according to the *Guide for the Care and Use of Laboratory Animals*, as adopted by the National Institutes of Health, and with approval of the University of California, San Francisco, Institutional Animal Care and Use Committee.

### Behavioral testing

Learning and memory were assessed with the Morris water maze (MWM) test (75). Briefly, 12 sessions of visible platform training were performed prior to hidden platform training as a control. Subsequently, mice underwent two sessions of hidden platform training separated by a 2 h intersession rest. Each session consisted of two trials. This training was performed each day for 7 days. The platform was removed and memory probe trials were performed 24 h and 72 h after the last training day.

EthoVision video-tracking system (Noldus, Netherlands) was used to record and track mice. Open field locomotor activity was performed as described (20). Briefly, mice were habituated for at least 1 h before recording activity for 15 min with an automated Flex-Field/Open Field Photobeam Activity System (San Diego Instruments, San Diego, CA). All behavioral experiments were performed with the examiner blinded to genotype.

### Histology and immunocytochemistry

For histology experiments, mice were anaesthetized and perfused with phosphate-buffered saline (PBS) and then 4% paraformaldehyde (PFA). Brains were then removed, postfixed in PFA overnight, and cryoprotected in 30% sucrose. Coronal brain slices (30 μm) were prepared using a sliding microtome (Leica SM2000 R).

For immunocytochemistry experiments, neuronal cultures were prepared as described below on coverslips and fixed in 4% PFA for 20 min.

For immunofluorescence, sections and coverslips were blocked for ≥1 h in PBS with 0.2% or 0.5% (for 82E1) Triton X-100 and 5–10% bovine calf serum and then incubated with primary antibodies overnight at RT. The following primary antibodies were used: chicken anti-MAP2 (1:1000; Abcam Cat# ab5392, RRID:AB_2138153); mouse anti-NeuN (1:1000; Millipore Cat# MAB377, RRID:AB_2298772); rabbit anti-Tom20 (1:500; Santa Cruz, Cat# SC-11415, RRID:AB_2207533); mouse anti-PDH (1:400 and 1:1000, Abcam Cat#110333, RRID:AB_10862029); rabbit anti-Hsp60 (1:200, Proteintech Cat#15282-1-AP, RRID:AB_2121440); rabbit anti-MCU (1:200, Cell Signaling Cat#14997, RRID:AB_2721812); mouse anti-MAP2 (1:1000; Millipore Cat# MAB3418, RRID:AB_94856); rabbit anti-calbindin (1:20,000; Swant Cat# 300, RRID:AB_10000347); rabbit anti-APP (for mouse and human APP; 1:200; Abcam Cat# ab32136, RRID:AB_2289606); mouse anti-8E5 (for human APP; 1:5000 (37)); rabbit anti-sigma-1 receptor (1:200, Abcam Cat#Ab53852 (47)). Sections and coverslips were rinsed and incubated for 2 h at RT with the corresponding secondary antibodies: Alexa Fluor or DyLight 350, 488, 594, or 647 anti-mouse, chicken, or rabbit IgG (1:100–1:500; Invitrogen). For peroxidase staining, sections were quenched with 3% H_2_0_2_ and 10% methanol in PBS and blocked in 10% bovine calf serum and 0.2% gelatin in PBS with 0.5% Triton X-100. They were incubated with mouse anti-82E1 (1:1000; IBL - America (Immuno-Biological Laboratories) Cat# 10,326 RRID:AB_10705565), followed by biotinylated goat anti-mouse IgG (1:300; Vector Laboratories, Burlingame, CA; BA-1000, RRID:AB_2313606), and subsequently streptavidin-conjugated horseradish peroxidase (HRP) (1:300; Vectastain ABC kit, Vector Laboratories). Immunostaining was visualized with hydrogen peroxide and 3,3'-diaminobenzidine (DAB, Sigma).

Brain sections and coverslips were imaged with a laser-scanning confocal microscope (Zeiss LSM510-Meta, Zeiss LSM780-NLO FLIM, or Leica TCS SP8X) with a 63x (1.4 NA) PlanApo oil objective or (1.2 NA) C-Apochromat water objective, a Nikon Ti-E inverted microscope with a 60x (1.2 NA) PlanApo water objective, or a Keyence inverted microscope BZ-9000 with a 10x (0.45 NA) CFI PlanApo λ objective. Volume was calculated with the Cavalieri principle (76). Quantification of fluorescence and area was performed blind to genotype with MetaMorph software (version 7.7.3.0; Universal Imaging, RRID:SciRes_000136). Neuronal density was calculated by dividing the total fluorescence of NeuN in 100 μm^2^ by the average NeuN intensity per CA1 neuron. Quantification of cells with swollen mitochondria was scored blind to genotype, based on the presence of three or more swollen mitochondria in a cell (a subjective criterion chosen to distinguish Drp1cKO versus control mitochondria). Amyloid plaque load was calculated based on % area of the hippocampus covered by plaques. Colocalization of MAM images was analyzed using Imaris software and the surface–surface colocalization extension.

### Neuronal culture and live imaging

Postnatal hippocampal neuronal cultures were prepared from P0 Drp1^lox/lox^ mice as described (20) and transfected *via* electroporation (Amaxa) with one or more of the following constructs, all expressed in the pCAGGS vector downstream of the chicken actin promoter (77): ATP-YEMK (kind gift of Dr Noji, Osaka University) (55), mCherry-synaptophysin (78), Cre recombinase (20), hAPP mutant (Swedish, Indiana) (41, 42), ires-mApple, mitoGFP (5), GCaMP6f (51), Cepia3mt (43), R-CEPIA1er (41), ER-eYFP (Clontech), or mitoFarRed. mitoFarRed was generated by fusing TagRFP657 (kind gift from Vladislav Verkhusha (Albert Einstein)) to the mitochondria-targeting sequence, cytochrome C oxidase subunit VIII (79, 80). Neurons were cultured for 8–11 days before live imaging or analysis.

Live imaging was performed in Tyrode’s medium (pH 7.4; 127 mM NaCl, 10 mM HEPES-NaOH, 2.5 mM KCl, 2 mM MgCl_2_, 2 mM CaCl_2_, with or without 30 mM glucose and/or 10 mM pyruvate) on a Nikon Ti-E inverted microscope with an iXon EMCCD camera (Andor Technology) and a perfusion valve control system (VC-8, Warner Instruments) controlled by MetaMorph Software. Live imaging for MAM studies was performed on a Zeiss LSM880 confocal microscope with Airyscan detector. Field stimulations (10 Hz∗60 s and 30 Hz∗3 s) were performed with an A385 current isolator and a SYS-A310 accupulser signal generator (World Precision Instruments). Glycolysis was inhibited with 2-DG (5 mM, Sigma-Aldrich) and iodoacetate (1 mM, Sigma-Aldrich). Respiration was inhibited with oligomycin (3 μM). ER calcium efflux was stimulated with caffeine (25 mM, Sigma-Aldrich).

For GCaMP6f and Cepia3mt calcium experiments, images were obtained (490/20 ex, 535/50 em, Chroma) every 200 msec, while for R-CEPIA1er experiments, images were obtained (572/35 ex, 632/60 em, Chroma) every 500 msec. A region of interest was drawn over the cell body, excluding the nucleus, and the background-subtracted fluorescence was calculated for each timepoint, normalized to the baseline level of background-subtracted fluorescence and control.

For live MAM quantification, three-dimensional reconstructions of confocal Z-stacks (rendered via max projection) were created showing neuronal cell bodies with MAMs identified by areas showing ER-mitochondria marker colocalization (50).

For FRET experiments, sequential images were taken in the CFP (430/24 ex, 470/24 em), YFP (500/20 ex, 535/30 em), and FRET channels (430/24 ex, 535/30 em) with an ET ECFP/EYFP filter set (Chroma). Synaptic boutons were identified based on morphology. The FRET/donor ratio was calculated for each bouton and cell body as described (81), where FRET = (I_FRETCFP_∗BT_CFP_ – I_YFP_∗BT_YFP_)/I_CFP_, such that I_X_ is the background-corrected fluorescence intensity measured in a given channel. BT_CFP_ (donor bleed through) and BT_YFP_ (direct excitation of the acceptor) were calculated by expressing CFP and YFP individually and determining the ratios of I_FRET_/I_CFP_ and I_FRET_/I_YFP_, respectively.

### Electron microscopy

For EM of hippocampal neurons, DIV10 cultures were fixed in 4% PFA for 2 h and then incubated with 3% glutaraldehyde and 1% PFA in 0.1 M sodium cacodylate buffer (pH 7.4) overnight. Following the fixation, the cultures were processed through 2% osmium tetroxide and 4% uranyl acetate, then dehydrated and embedded in Eponate 12 resin (Ted Pella Inc, Redding, CA). Ultrathin sections were cut at 1-μm thick, collected on copper grids, and imaged in a Phillips Tecnai10 transmission electron microscope at an operating voltage of 80 kV using FEI software. Quantitative analysis was performed on digital EM images obtained with a charge-coupled device (CCD) camera at a final magnification of 11,500 (20). The quantification of EM samples from cultured mouse hippocampal neurons was performed with the examiner blinded to the genotypes using MetaMorph software (version 7.7.3.0; Universal Imaging, RRID: SciRes_000136).

### Statistical analysis of Morris water maze

MWM data has several characteristics that make longitudinal analysis complex: the data typically contain censoring, as the mice are removed from the water after a fixed amount of time if they fail to complete the task, the learning effect is often very nonlinear; as healthy mice often learn the maze as well as they can before the last trial and thus stop systematically improving, there is typically a learning effect of both days of trials and number of trials given that day, which leads to a “saw-tooth” learning effect, and finally, a mean-variance relation is expected.

Rather than attempting to build a very complex statistical model to account for these data features, a summary measure analysis (82) was created, which greatly reduced the dimensionality of the problem and allowed for simple, robust, powerful, and easily interpreted results. To do this, at each trial, each mouse is ranked (*i.e.*, which mouse finished first, second, etc.). Mice that failed to locate the platform are considered “tied for last.” For each mouse, the average rank across all trials is then calculated. This simple composite score is used in standard analyses.

The outcomes considered were the average ranks of latency per mouse during hidden and visible trials. The data were fit to two linear mixed-effects models (83) corresponding to these outcomes and to the factor genotype using the R package lme4 (RRID:nif-0000-10474) (84). Random effects for the effect of cohort on genotype were included, and the overall effect of genotype on average rank latency hidden and average rank latency visible was tested using the Wald Chi-square test.

The fitted model was used to obtain estimates of the mean difference in ranks. The function sim() from the arm package (85) yielded 50,000 draws of the group effects, from which 95% confidence interval (CI) around each estimate as the 2.5th and 97.5th quantiles was calculated. *p*-values for differences between groups were calculated using the simulated differences. The *p*-values corresponding to the composite scores were not adjusted as the gatekeeping testing approach was appropriate to use here (86, 87). The *p*-values corresponding to the rest of the outcomes were corrected for multiple comparisons using the method of Holm (88).

For the memory probe trials, two different outcomes were analyzed: latency to first target platform crossing and number of platform crossings. Data were fit onto a Cox proportional hazards regression model of latency on genotype using the R package survival (89). The proportional hazards assumption was examined by visual inspection of the curves of the natural logarithm of the cumulative hazard function versus latency for each of the four genotypes. The four curves were approximately parallel, indicating that the proportional hazards assumption was met for this data set. Kaplan–Meier nonparametric test was also performed to investigate the survival function for each genotype. Random effects for the effect of cohort on genotype were included.

The number of platform crossings by genotype data were fit to a Quasi-Poisson generalized linear model. The Quasi-Poisson generalized linear model accounts for overdispersion, allowing for more robust estimation. Two Quasi-Poisson models were used, one with an interaction term between genotype and cohort, to ensure that the treatment effect was relatively constant across cohorts, and the primary model was fit with only genotype alone (assuming the effect of genotype was consistent across cohorts). A deviance test was used to compare these two models, revealing no significant difference between them (*p*-value=0.166), implying that the effects were consistent across cohorts. Therefore, the model with only the effect of genotype was used.

Estimates of the relative risk of reaching the platform (RR), the 95% confidence interval of the relative risk, and the corresponding *p*-values were obtained from the Cox proportional hazards regression model. The mean difference in number of platform crossing, the 95% confidence interval of the difference, and the corresponding *p*-values were obtained from the Quasi-Poisson model. The *p*-values corresponding to these outcomes were corrected for multiple comparisons using the method of Holm (88).

## Data availability

Data described in the article is available upon request. Please contact Ken Nakamura: Ken Nakamura, MD, PhD, Gladstone Institute of Neurological Disease, 1650 Owens Street, San Francisco, CA 94158, Phone: (415) 734-2550; Fax: (415) 355-0824; E-mail: ken.nakamura@gladstone.ucsf.edu

## Supporting information

This article contains supporting information.

## Conflict of interest

The authors declare that they have no conflicts of interest with the contents of this article.
