# Supplementary material for: Mitochondrial fission is a critical modulator of mutant APP-induced neural toxicity
Source: J Biol Chem. 2021 Feb 25;296:100469. doi: 10.1016/j.jbc.2021.100469 (PMC8042169; doi:10.1016/j.jbc.2021.100469)
Supplement: Figures S1–S9 [file mmc1.pdf]

SUPPORTING FIGURES AND FIGURE LEGENDS

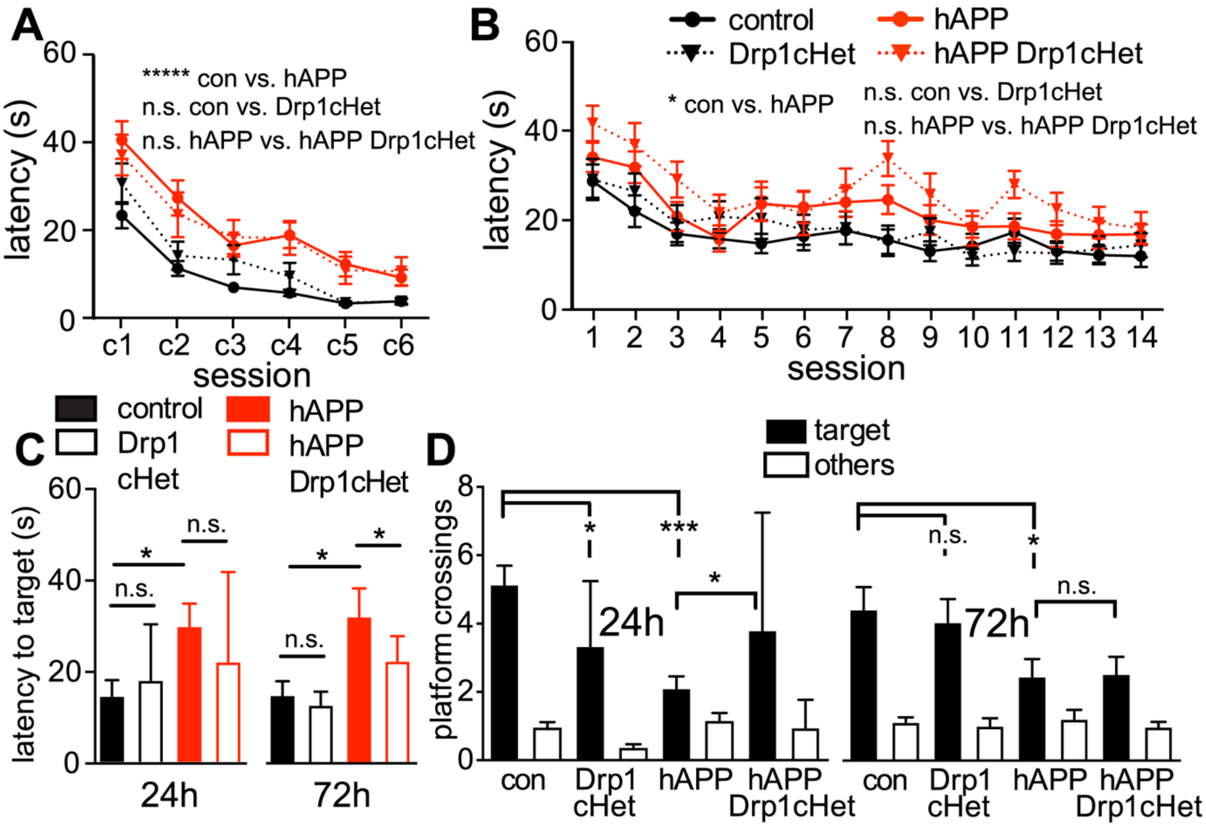

Figure S1. Drp1 heterozygosity may protect against memory deficits in hAPP mice.
A,B, Drp1cHet (Drp1$^{wt/lox}$;CamKII-Cre) and hAPP Drp1cHet (hAPP-J20;Drp1$^{wt/lox}$;CamKII-Cre) mice compared to wild-type and hAPP (hAPP-J20;Drp1$^{wt/lox}$ and hAPP-J20;Drp1$^{lox/lox}$) did not exhibit differences in procedural cued training nor memory during hidden-platform training in 6–7-month-old mice over 14 sessions (7 days), Data are means ± S.E.M.; n.s. = not significant, *p<0.05, *****p<0.00001, by average rank latency with mixed effect modeling, n=15–22 mice/group. *C*, Spatial memory was evaluated using MWM probe trials at 24 and 72 h with the hidden-platform removed, and measured by latency to cross the former hidden platform location (target) and *D*, number of target and non-target (other) platform location crossings. hAPP mice showed significant memory deficits that were partially blocked by Drp1 heterozygosity. n=15-22 mice/group. Data are means ± S.E.M.; n.s. = not significant, *p<0.05, ***p<0.001 by Cox proportional hazards regression models (latency to cross) and Quasi-Poisson generalized linear models (platform crossings). Wild-type and hAPP data presented in this figure are also shown in Figure 1, and all mice in Figure 1 and S1 were evaluated in the same experiment, but the figures are separated for clarity.

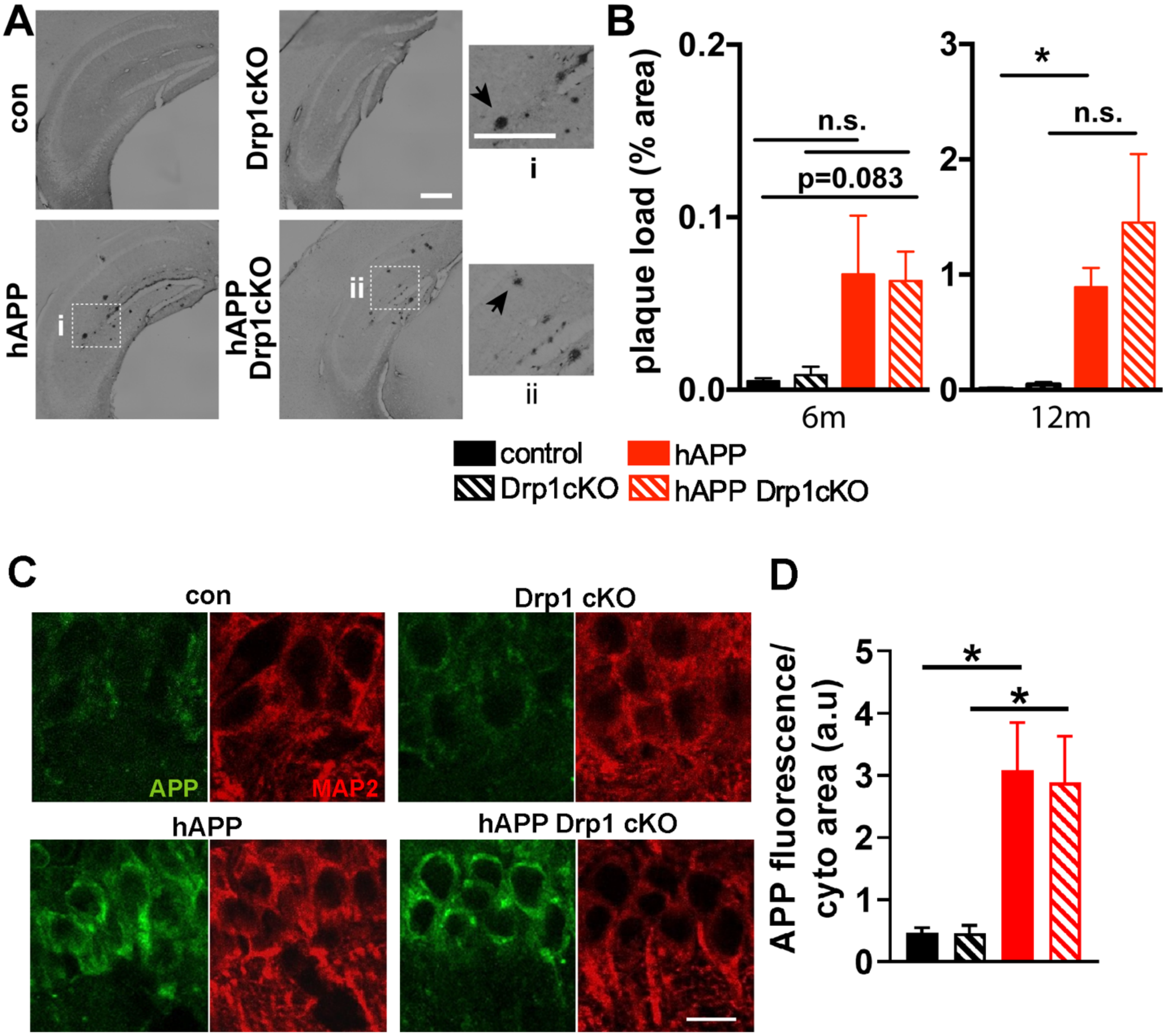

Figure S2. Drp1cKO does not impact amyloid beta deposition or APP levels.

A, Aβ deposits in hippocampi of 12-month-old hAPP mice with or without Drp1, identified by immunohistochemistry (82E1 antibody). Scale bar: 400 µm. *B*, hAPP expression led to Aβ deposition at 6 and 12 months, although comparisons did not reach significance until 12 months. Drp1cKO had no impact on Aβ deposition. Data are means ± S.E.M.; n=4–5 mice/group (with 3 slices of hippocampus examined per mouse); n.s. = not significant, p=0.083, *p<0.05 by Welch's ANOVA and Games-Howell *post hoc* test (used instead of two-way ANOVA due to significant Levene's test for equality of variance). *C*, Immunostaining for APP in CA1 neurons in hippocampal slices from 6-7- month old Drp1WT (control), Drp1cKO, hAPP-J20 (hAPP), and hAPP-J20 Drp1cKO (hAPP Drp1cKO) mice. Cell bodies were defined by MAP2 staining. *D*, Quantification of APP staining. n=4 mice/group (2-3 slices/mouse, 41- 43 cells/group). Data show mean ± SEM; p=0.0238, p=0.0356, *p<0.05, by two-way ANOVA and Holm-Sidak's multiple comparisons test. The scale bar is 30µm.

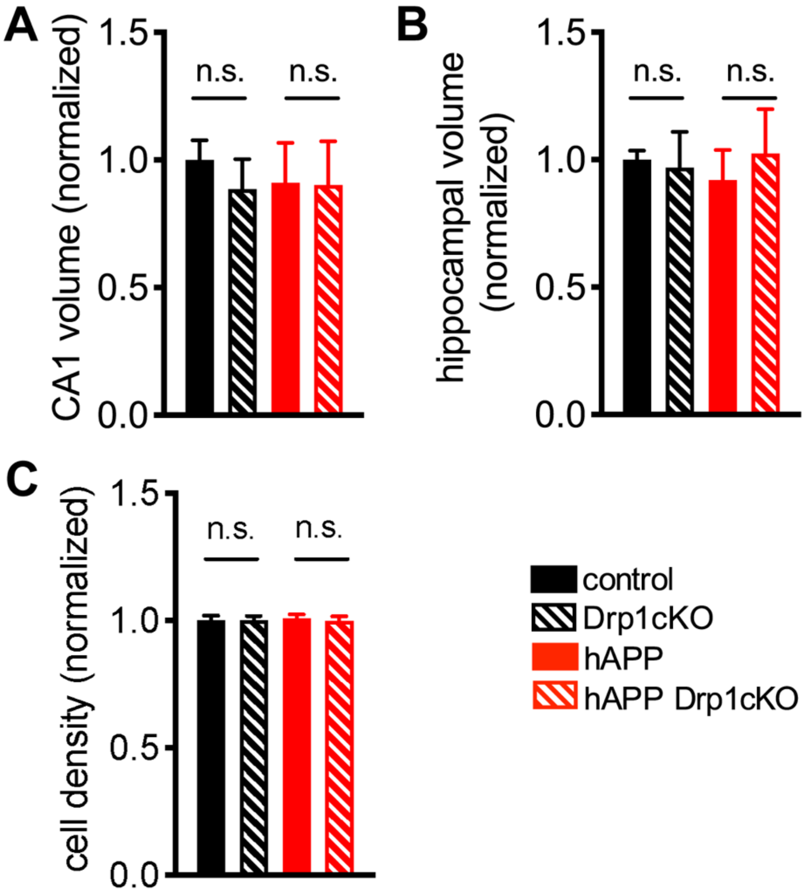

Figure S3. hAPP Drp1cKO does not cause cell loss in vivo at 6 months.
A,B, CA1 (A) and overall hippocampal (B) volume were similar between groups in 6-month-old mice. n=4 mice/group (4–8 slices/mouse). *C*, Drp1cKO and hAPP Drp1cKO mice did not show any decrease in CA1 cell density at 6 months of age. Data are means ± S.E.M.; n=4 mice/group (4 slices/mouse). n.s. (not significant) by two-way ANOVA and Holm-Sidak *post hoc* test.

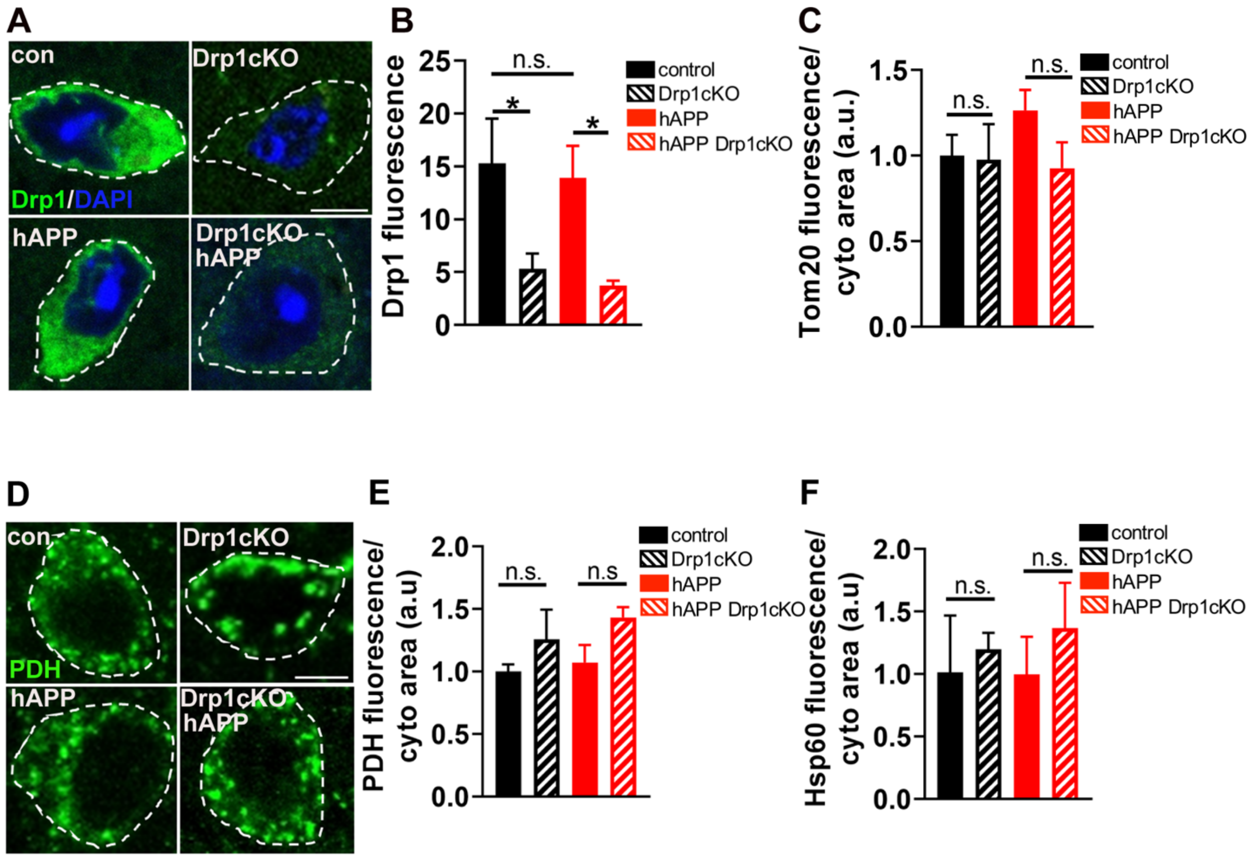

Figure S4. hAPP does not impact mitochondrial content in hAPP Drp1cKO neurons.
A,B, Mean Drp1 fluorescence in the cytoplasm of CA1 neurons in hippocampal slices from 6-7- month old Drp1WT (control), Drp1cKO, hAPP-J20 (hAPP), and hAPP-J20 Drp1cKO (hAPP Drp1cKO) mice. *C-F,* Mitochondria in CA1 neurons identified by the outer mitochondrial membrane protein Tom20 (C, see also Fig. 2E), and the mitochondrial matrix proteins PDH (montage in D, E) and HSP60 (F). Quantification revealed no differences between groups in the mean cytoplasmic fluorescence of any of the mitochondrial markers of CA1 cells at 6 months. Cell bodies (outer stippled outlines) were defined by MAP2 staining. n=4 mice/group (A, 2-3 slices per mouse, 16-19 cells/group; C,E,F, 3-4 slices per mouse, 50-69 cells/group). n.s. (not significant) by two-way ANOVA and Holm-Sidak *post hoc* test. The scale bar is 5 µm.

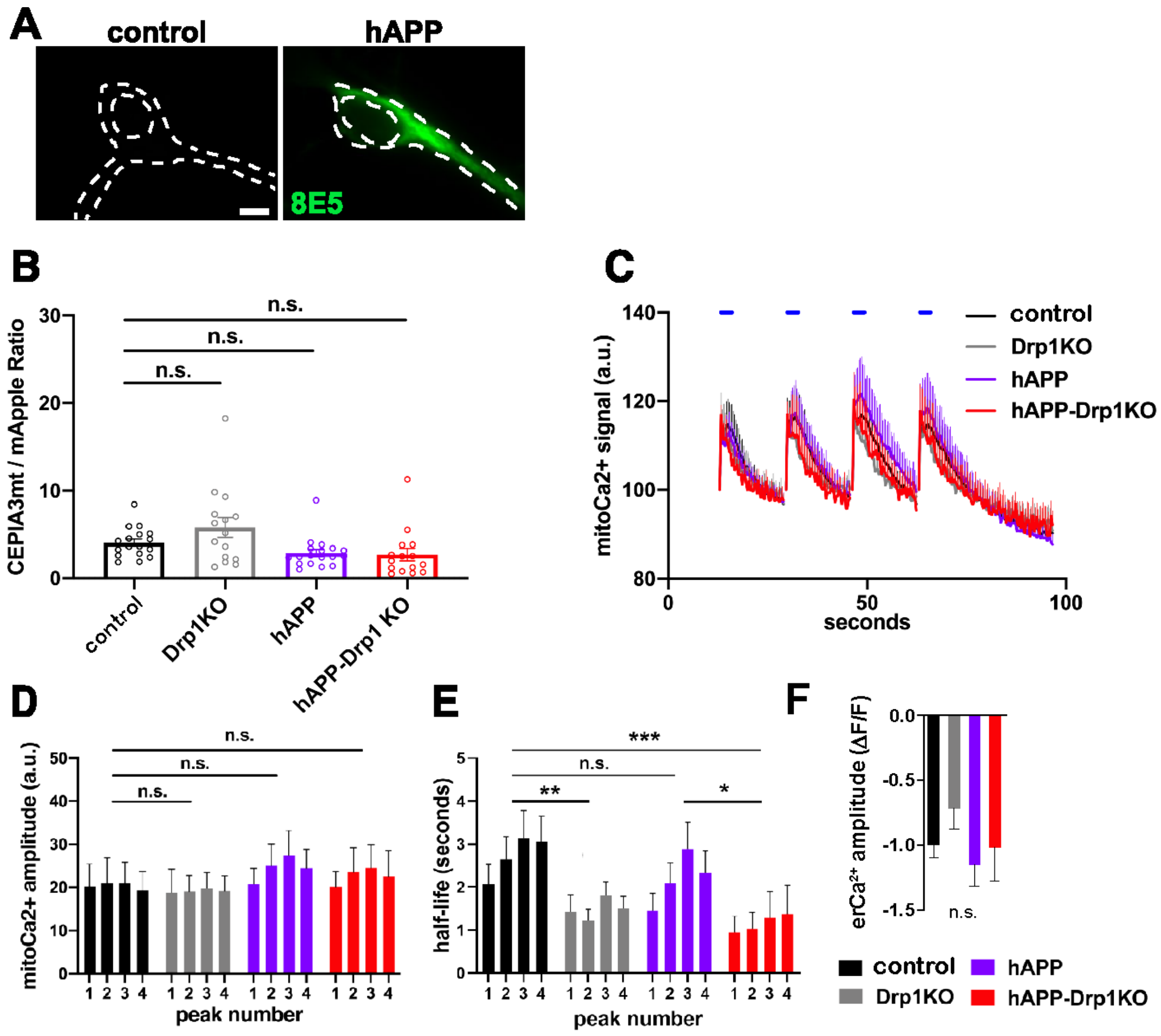

Figure S5. Mitochondrial calcium dynamics of neurons that recover mitoCa^{2+} following stimulation. *A*, Drp1$^{lox/lox}$ hippocampal neurons transfected with hAPP express the protein, as assessed by the 8E5 antibody, which is specific to human APP. Scale bar is 5 µm. *B-D*, Drp1KO and control neurons were co-transfected with the mitoCa^{2+} sensor CEPIA3mt, mApple, and hAPP or its control vector, and were subjected to a sequence of four individual electrical stimuli (30Hz for 3s, blue horizontal lines) to evoke calcium entry. *B*, Basal mitoCa^{2+} level estimated by the ratio of fluorescence intensity of CEPIA3mt to mApple. There are no differences between the three experimental groups and control. n=15-18 coverslips (1 cell/coverslip), compilation of 6 independent experiments. Data show mean ± SEM; not significant (n.s.) by Welch's ANOVA test and Games-Howell multiple comparison test. *C*, Average mitoCa^{2+} levels for neurons that successfully recovered mitoCa^{2+} following each stimulation for control (black), Drp1KO (grey), hAPP (purple) and hAPP-Drp1KO (red) conditions. *D*, Average amplitude for each mitoCa^{2+} peak in (C). All groups exhibited similar mitoCa^{2+} loading during electrically-evoked calcium entry as control. *E*, Average half-life in seconds for each mitoCa^{2+} peak in (C). Drp1KO alone or in combination with hAPP increases mitoCa^{2+} efflux rate, while hAPP alone has no impact on half-life. n=8-16 coverslips/group (1 cell/coverslip), compilation of 6 independent experiments. *C-E*, Data show mean ± SEM; n.s. (not significant), *p<0.05, **p<0.01, ***p<0.001 by two-way repeated measures ANOVA and Holm-Sidak *post*

hoc test. *F*, Drp1KO neurons with or without mutant hAPP were co-transfected with the ER-targeted calcium sensor R-CEPIA1er ***(45)***. ER calcium was measured while stimulating release (measured as a decrease in calcium) with caffeine (25 mM). Neither hAPP nor Drp1KO significantly affected the magnitude of ER calcium release. n=8–10 coverslips/group (with 11–17 cells/group). Data are means ± S.E.M; comparisons are not significant (n.s.) by one-way ANOVA and Holm-Sidak *post hoc* test.

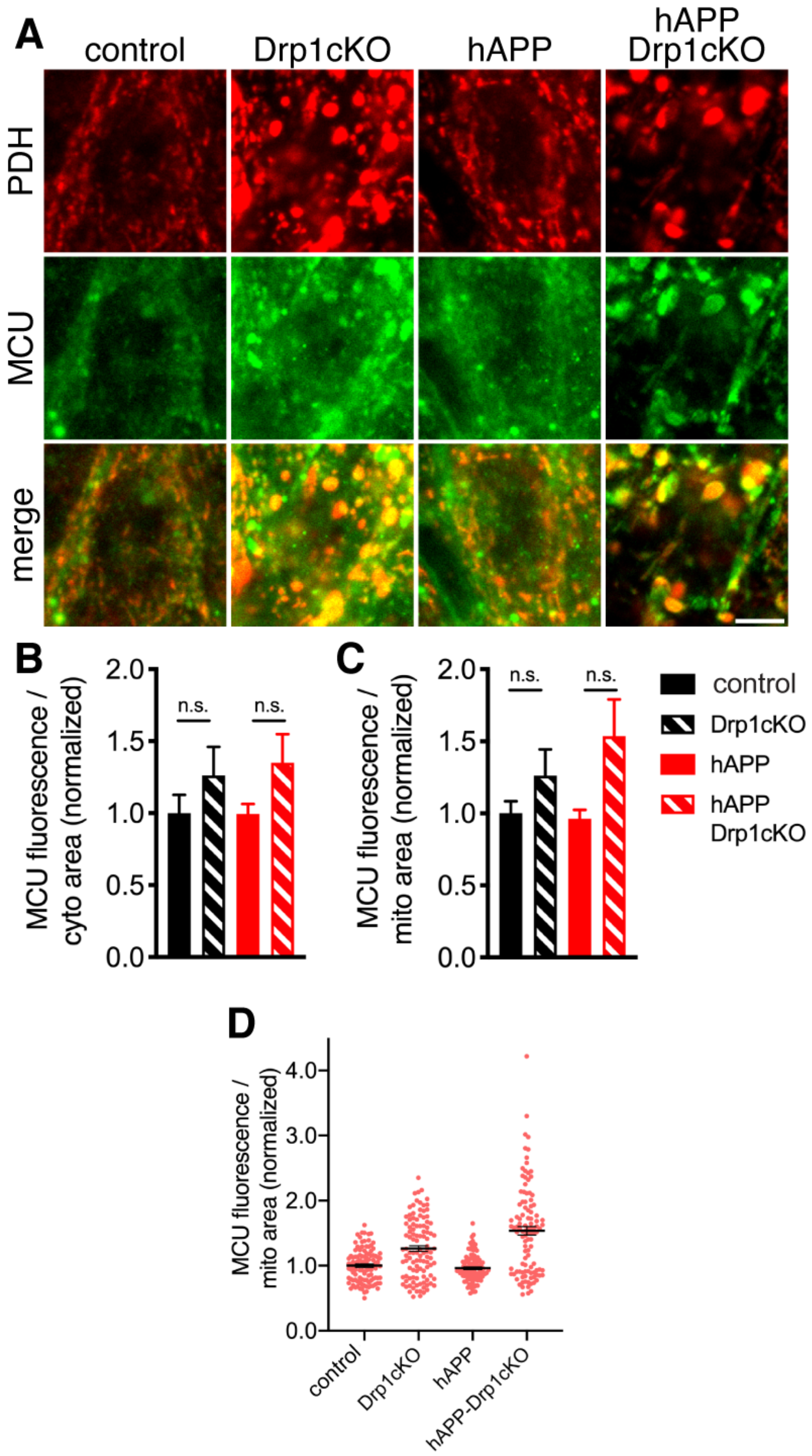
A
control
Drp1cKO
hAPP
hAPP
Drp1cKO
PDH
MCU
merge
B
MCU fluorescence /
cyto area (normalized)
2.0
1.5
1.0
0.5
0.0
n.s.
n.s.
C
MCU fluorescence /
mito area (normalized)
2.0
1.5
1.0
0.5
0.0
n.s.
n.s.
control
Drp1cKO
hAPP
hAPP
Drp1cKO
D
MCU fluorescence /
mito area (normalized)
4.0
3.0
2.0
1.0
0.0
control
Drp1cKO
hAPP
hAPP-Drp1cKO

Figure S6. Drp1KO and hAPP do not affect MCU expression in hippocampal neurons.
A, CA1 hippocampal neurons from 6-7- month old Drp1WT (control), Drp1cKO, hAPP-J20 (hAPP), and hAPP-J20 Drp1cKO (hAPP Drp1cKO) mice immunostained for MCU (green) and PDH (red). Scale bar is 5 µm. *B,C*, Quantitation revealed no differences between groups in the mean cytoplasmic fluorescence of MCU (B), or in the MCU fluorescence intensity specifically in mitochondria, identified by overlap with PDH staining (C). *D*, Graph shows individual data points (cells) from analysis performed in (C). Data are means ± S.E.M.; n = 6 sections/group (B, 2 sections/mouse, 6 cells/section; C, 2 sections/mouse, 18 cells/section). n.s. = not significant by Welch's ANOVA and Games-Howell *post-hoc* test.

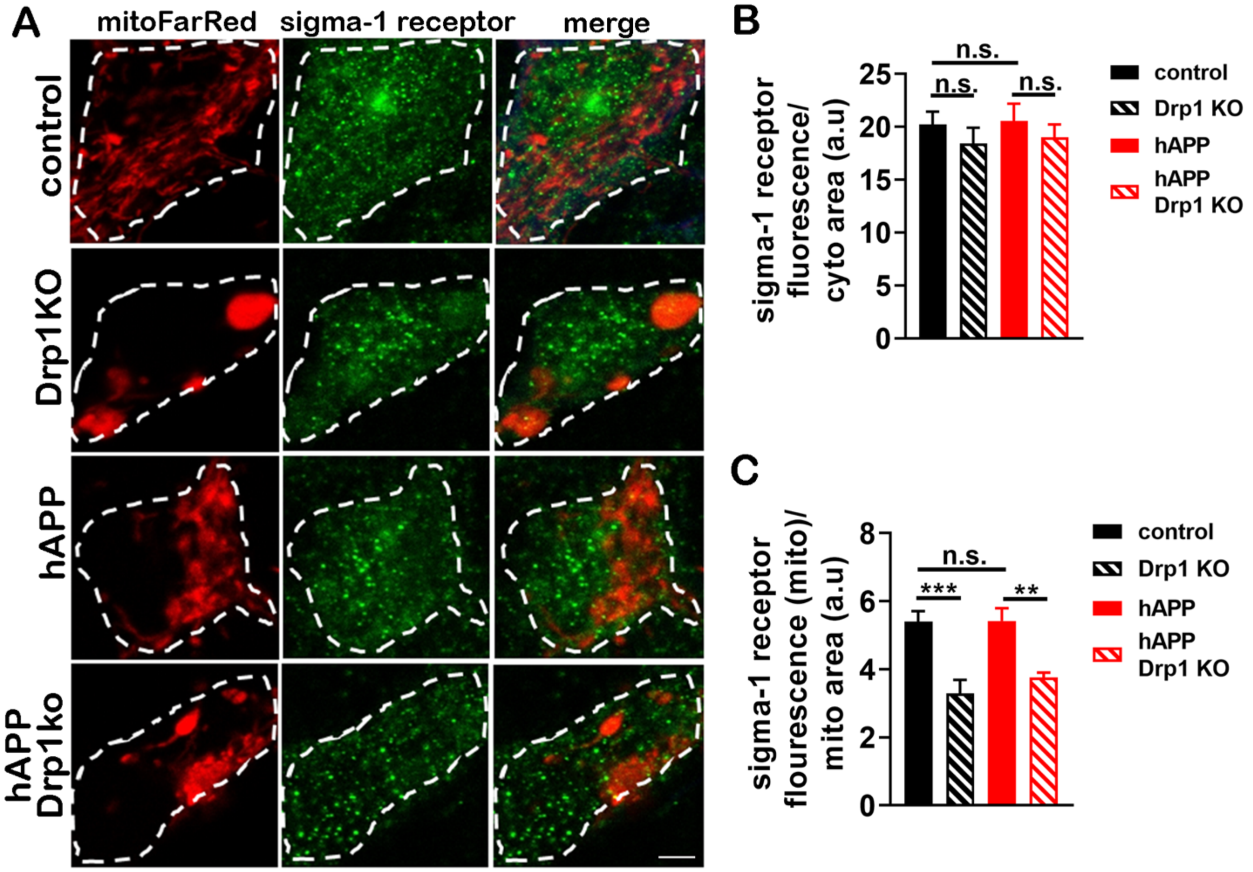

Figure S7. Drp1 loss decreases sigma-1 receptor co-localization with mitochondria in cultured neurons. Drp1KO and control neurons expressing mutant hAPP, and/or control vector (control), as well as mitoFarRed to visualize the mitochondria, were immunostained for sigma-1 receptor (green) and MAP2 (not shown) to define the margins of cell bodies (white stippled lines). Scale bar is 4 µm. *B*, Mean cytosolic fluorescence of sigma-1 receptor was unchanged between groups. *C*, However, both Drp1KO and hAPP Drp1KO had significantly decreased sigma-1 receptor fluorescence co-localizing with mitochondria. n= 16-24 cells/group from 4 coverslips. Data are means ± S.E.M.; n.s. = not significant, **$p<0.001$, ***$p<0.0001$ by two-way ANOVA and Holm-Sidak *post hoc* test.

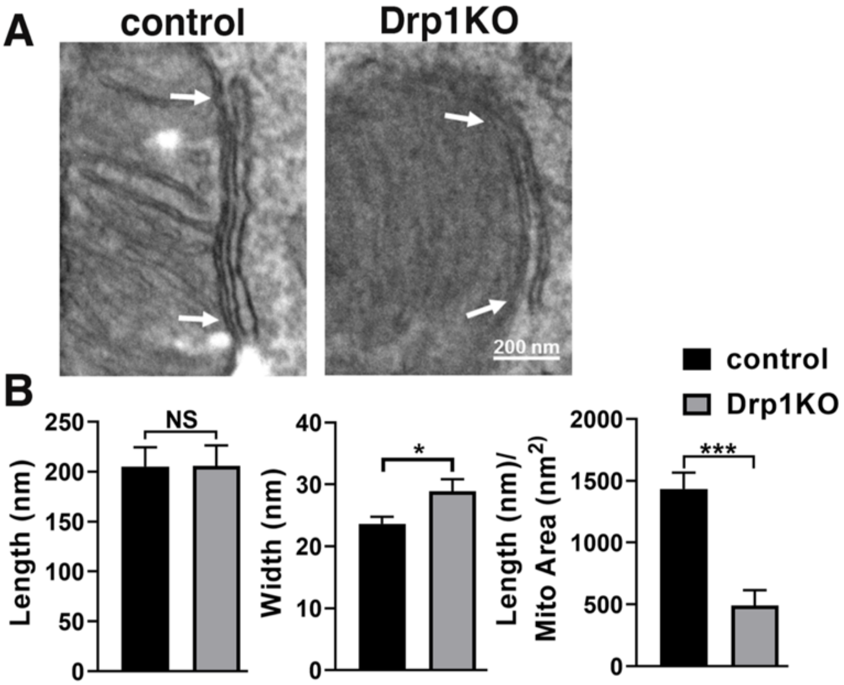

Figure S8. Drp1 loss decreases MAMs in neurons. *A,* EM images show representative MAMs (arrows) in the cell body of control and Drp1KO hippocampal neurons. MAMs are defined as <50 nm separation between the ER and mitochondria (white arrows). *B*, Drp1KO mitochondria have MAMs with similar length as controls. However, the width is increased, while the length of ER-mitochondria contact per mitochondrial area is markedly decreased in Drp1KO mitochondria. n=50 mitochondria in control, n=25 mitochondria in Drp1KO from 2 independent experiments. NS = not significant, *p<0.05, ***p<0.001 by unpaired t-test. Scale bar is 200nm.

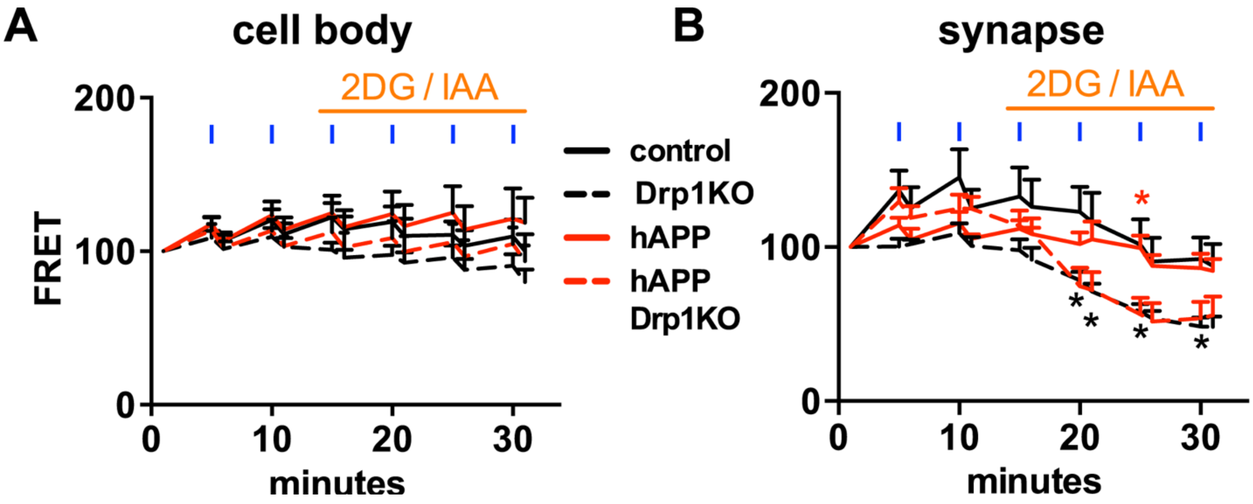

Figure S9. hAPP does not cause or accentuate Drp1KO-induced mitochondrial ATP deficits at the synapse. Drp1-floxed hippocampal neurons were co-transfected with an ATP-based FRET sensor (ATP1.03^{YEMK}) ***(82)***, and a control vector (control), Cre (Drp1KO), and/or mutant hAPP. *A, B*, To measure mitochondrial-derived ATP levels, glycolysis was blocked with 2-deoxyglucose (2DG) and iodoacetate (IAA, orange bar). Neurons were stimulated at 30 Hz*3 s (blue vertical bars). *A*, All groups maintained similar ATP levels observed at the cell body, with no significant differences between groups. *B*, Neurons lacking Drp1 were unable to maintain ATP levels at the synapse, but hAPP had no effect on ATP. Data are means ± S.E.M.; *$p<0.05$ control *versus* Drp1KO (in black) and hAPP *versus* hAPP Drp1KO (in red) by two-way ANOVA with repeated measures and Holm-Sidak *post hoc* test, n=7-11 coverslips/group (with 72-106 boutons and 9-14 cells per group).
